# A review of center of pressure (COP) variables to quantify standing balance in elderly people: Algorithms and open‐access code*

**DOI:** 10.14814/phy2.15067

**Published:** 2021-11-26

**Authors:** Flavien Quijoux, Alice Nicolaï, Ikram Chairi, Ioannis Bargiotas, Damien Ricard, Alain Yelnik, Laurent Oudre, François Bertin‐Hugault, Pierre‐Paul Vidal, Nicolas Vayatis, Stéphane Buffat, Julien Audiffren

**Affiliations:** ^1^ Centre Borelli UMR 9010/Université Paris‐Saclay ENS Paris‐Saclay CNRS SSA, Inserm Université de Paris Paris France; ^2^ ORPEA Group Puteaux France; ^3^ Groupe MSDA Université Mohammed VI Polytechnique Benguerir Maroc; ^4^ Service de Neurologie de l’Hôpital d’Instruction des Armées de Percy SSA Clamart France; ^5^ Ecole du Val‐de‐Grâce Ecole de Santé des Armées Paris France; ^6^ PRM Department GH Lariboisière F. Widal AP‐HP Université de Paris UMR 8257 Paris France; ^7^ Institute of Information and Control Hangzhou Dianzi University Zhejiang China; ^8^ Laboratoire d’accidentologie de biomécanique et du comportement des conducteurs GIE Psa Renault Groupes Nanterre France; ^9^ Department of Neuroscience University of Fribourg Fribourg Switzerland

**Keywords:** center of pressure, elderly, postural control, quiet standing

## Abstract

Postural control is often quantified by recording the trajectory of the center of pressure (COP)—also called stabilogram—during human quiet standing. This quantification has many important applications, such as the early detection of balance degradation to prevent falls, a crucial task whose relevance increases with the aging of the population. Due to the complexity of the quantification process, the analyses of sway patterns have been performed empirically using a number of variables, such as ellipse confidence area or mean velocity. This study reviews and compares a wide range of state‐of‐the‐art variables that are used to assess the risk of fall in elderly from a stabilogram. When appropriate, we discuss the hypothesis and mathematical assumptions that underlie these variables, and we propose a reproducible method to compute each of them. Additionally, we provide a statistical description of their behavior on two datasets recorded in two elderly populations and with different protocols, to hint at typical values of these variables. First, the balance of 133 elderly individuals, including 32 fallers, was measured on a relatively inexpensive, portable force platform (Wii Balance Board, Nintendo) with a 25‐s open‐eyes protocol. Second, the recordings of 76 elderly individuals, from an open access database commonly used to test static balance analyses, were used to compute the values of the variables on 60‐s eyes‐open recordings with a research laboratory standard force platform.

## Graphical abstract

The lack of description of calculation methods for the variables extracted from the stabilogram limits reproducibility and comparison between studies. We propose an explicit corpus of postural variables with their values computed on two databases.

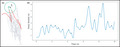

## 1 INTRODUCTION

The assessment of balance disorders is a common practice in geriatric care, as the problem of falls in the elderly is so serious in maintaining good health. As a major public health problem, falls are the leading cause of accidental death in the elderly, leading to serious psychomotor consequences and accelerating institutionalization (WHO, 2008). Health authorities recommend a standardized risk assessment for falls in the elderly that includes identification of risk factors and assessment of motor control. The latter is often carried out through functional clinical tests requiring the performance of one or more exercises while an operator assesses the feasibility of the task for the participant (Beauchet et al., 2011). Limitations of functional tests relate to the ability to distinguish the systems disturbed in relation to imbalance (vestibular, visual, proprioceptive, motor, etc.), the provision of quantified and objective values, as well as a capacity to discriminate between fallers that is both more effective than a history of past falls and sufficiently sensitive to the evolution of balance capacities in the short and medium term (Balasubramanian, 2015; da Costa et al., 2012; Mancini & Horak, 2010), especially in extended care settings where the risk of fall is higher.

To address the lack of reliable clinical tests in the evaluation of balance and posture disorders, posturography aims at developing quantifiable analyses of postural control (Baloh et al., 1998), mainly through the analysis of the trajectory of the center of pressure (COP). The COP trajectory is recorded using force platforms, which track the point of application of the ground reaction forces resultant under the feet. The resulting signal, called stabilogram, is frequently analyzed using either its one‐dimensional variations in the mediolateral (ML) or anteroposterior (AP) direction, or its two‐dimensional trajectory (de Sá Ferreira & Baracat, 2014; Duarte & Zatsiorsky, 2011). The COP signal is then described using a number of variables, which are used to evaluate the risk of fall. This approach has produced interesting results in the assessment of the risk of falling, in subjects with a balance degradation due to neurological impairment (Ojala et al., 1989; Vališ et al., 2012) or physiological aging (Baloh, Jacobson, Enrietto, et al., 1998; Camicioli et al., 1997; Colledge et al., 1994; Perrin et al., 1997). In quiet standing, the COP is considered to reflect in part the motor mechanisms that ensure balance, precisely the maintenance of the projection of the center of mass (COM) inside the base of support (Hof et al., 2005). There is a correlation between the displacement of the COP at the limits of stability and the incidence of falls, underlining the interest of exploring dynamic balance in determining the risk of falling (Johansson et al., 2019). In addition, time‐to‐boundaries analysis has revealed significant spatio‐temporal instabilities during voluntary excursion by leaning in all directions on force platform in elderly people compare to younger subjects (van Wegen et al., 2002). Nevertheless, a simple test to quantify resting balance on a firm and stable surface is thought to already provide relevant information for the analysis of fall risk (Bauer et al., 2016b; Lord & Clark, 1996). Thus, static posturography on a force platform could be a convenient tool for assessing the risk of falling, particularly for the oldest people for whom psychomotor disorders are known to exist and greatly limit the possibility of conducting functional tests that compromise their already precarious balance. The quantification of balance using a force platform is now commonly used (Pizzigalli et al., 2014).

Despite the relevance of exploring balance through quantified and explainable COP variables for the clinicians, their computation suffers from significant drawbacks. First, studies may present different definitions of the same variable or may not give a precise definition. For example, several variables rely on the calculation of peaks in particular signals obtained from the COP, however the method used for calculating these peaks is not explicitly defined, evoking notions of maximum values (Doyle et al., 2005) or high values between two “valleys” (Baratto et al., 2002) without any clear indication of a threshold in time or amplitude, and no clear algorithmic procedure. Moreover, the vocabulary used to introduce the variables sometimes varies from one study to another, making the identification of variables difficult, especially given that the equations used to calculate them are rarely provided. Second, the definitions of many of the COP variables rely on mathematical assumptions that are in general not clearly stated or verified (such as uniform resampling, see e.g. Audiffren & Contal, 2016). This lack of clarity can lead to contradictory conclusions between studies for the same variables (Delignières et al., 2011). Finally, even when clear computation procedures have been presented in the literature, some of them include several algorithmic steps which may not be convenient to code in the context of clinical practice (Chiari et al., 2000; Collins & De Luca, 1993), highlighting the need of developing open‐access codes to compute the variables. The aforementioned drawbacks make it particularly delicate to compare the results of different studies and generalize their finding.

The number of available variables in the literature is also challenging. Indeed, in a previous systematic review (Quijoux et al., 2020), we identified more than 50 variables derived from the trajectory of the COP recorded in quiet stance to discriminate elderly fallers from non‐fallers. A large number of these posturographic variables can be calculated along the AP or ML direction and in the two‐dimensional signal which further increases the quantity of variables that can be considered, leading to statistical problems related to data dimensionality. Moreover, since the semiological understanding of posture disorders is relatively limited, no consensus has been reached regarding the grouping of these variables under large physiological classes that could alleviate this problem—as may have been the case with gait (Mansour et al., 2017; Vienne et al., 2017).

The objective of this review is to propose a compendium of definitions of the COP variables that are the most frequently found in the literature to compare elderly fallers from elderly non‐fallers, based on a systematic review (Quijoux et al., 2020). The lack of standardized methods and analysis procedures has been proposed to explain discrepancies of results with similar analysis (Kirchner et al., 2012). Accordingly, we aim to facilitate the comparison between studies through a review of the scientific literature as well as the computation and the presentation of the values for the selected variables. The method of variable selection is presented below. In accordance with our selection process (see Section 2.1 and 2.2), we did not include in this review several postural control models and variables (Hernandez et al., 2015; Hur et al., 2012; Reed et al., 2020; Sakanaka et al., 2016), that were not used in the clinical examination of elderly people at risk of falling. Additionally, and to help the identification and comprehension of the variables, we also propose a new classification that reflects the aspects of the COP trajectories they are designed to capture: positional, dynamic, frequency, and stochastic variables. We hope that by providing this compendium, future works may compare and aggregate more easily their results. Furthermore, and to help the use of these variables, we propose a descriptive analysis of their behavior on two databases of COP trajectories recorded in elderly people. We provide the average values and standard deviation of each variable on both datasets, in order to provide a baseline for typical values or order of magnitudes that can be expected for these variables in an elderly population. Note that these datasets present a large variability of medical profiles, and have been collected with different protocols and equipments, thus hinting at the general scope of the indexed variables. The contribution of these two datasets is to present values from the same calculation methods, but for different experimental conditions, which we hope will provide a means of comparison for future users of these algorithms.

## 2 METHOD

### 2.1 Literature review

A systematic review of the literature was originally conducted to identify articles that addressed the discrimination of older people at risk of falling. Randomized control trials (RCTs), non‐randomized control trials, and observational studies were all eligible for inclusion. Articles analyzing the balance through COP recordings during quiet standing with both feet on the ground and evaluating the risk of falling by the number of falls during a period of time (retrospectively or prospectively) were selected. Four databases (PubMed, Cochrane CENTRAL, EMBASE, and ScienceDirect) were used as sources for published articles. The search was performed for all articles published (without date restriction) until July 1, 2019. In addition, a search of “grey” literature (Conn et al., 2003) was performed which included items like reports, theses, and studies that were found online using Google Scholar, ClinicalTrials.gov sources, Google, theses.fr, HAL, ResearchGates, and ethos.bl.uk. All reference lists from included studies were reviewed for additional relevant studies. The papers had to be written in a language understood by the authors (i.e. English, French, Italian, Spanish, or German). The choice was made to include a wide range of study types and not to limit the study to RCT in order to have a broad view of the COP analysis methods used to differentiate between fallers and non‐fallers of 60 years and older (Quijoux et al., 2019).

Studies, and the variables extracted from them, were included if the research involved a comparison of older people with and without a history of falls (retrospective studies) or longitudinal follow‐ups of these elderly people with regular measurement of the number of falls (prospective studies). Analyses of COP trajectories should be clearly stated, as well as the protocol for recording balance, excluding recordings of dynamic balance with instructions such as bending forward, repositioning after destabilization, or standing on one foot. It is of practical interest for balance analysis to distinguish between older people on the basis of their number of falls. Many studies have shown differences between healthy and young subjects compared to the elderly (Condron et al., 2002; King et al., 2016; Pizzigalli et al., 2014), but from a clinical point of view it seems more relevant to focus on the studies comparing individuals of the same age group.

### 2.2 Selection of COP variables

The variables presented in this work were selected as follows. Based on Quijoux et al. (2020)—a recent systematic review of the COP characteristics that were used to identify fall risk in elderly—and the aforementioned criterion, we identified 27 articles presenting results using measurements derived from the COP trajectory. Among the variables introduced in those articles, we selected all those that satisfied the following inclusion criteria:

- Must be used in at least two different articles
- Must be tested to distinguish older people at risk of falling from a control group, even if the variable is not discriminating
- Must be sufficiently described, with enough details, to be reproducible. This includes formal mathematical definition such as equations or explanations of computation methods.

It should be noted that for some variables included in this study, the description in previous works was only partial. In this case, additional hypotheses were made to permit the computation of the feature, and these assumptions are clearly stated in the paragraphs of this study dedicated to the sway variables concerned.

### 2.3 Corpus of the selected variables

Each variable is presented with references to its computation in previous studies and the algorithm that enables its calculation. The variables are grouped in four families to ease the reading of this study, according to their reliance on different aspects of the COP trajectories:

- Positional variables
  - Variables that describe characteristics of the dispersion of the trajectory or position of the feet, and do not require the knowledge of the dynamics of the signal.
- Dynamic variables
  - Variables based on the dynamic of the COP, requiring the knowledge of its local displacements.
- Frequency variables
  - Variables used to describe the power spectral density of the COP trajectory.
- Stochastic variables
  - Variables derived from the models in which the COP is represented as a stochastic process.

A more detailed description of each group is provided at the beginning of its respective part, in Section 3. It is important to note that these categories are not necessarily orthogonal, in the sense that features inside different groups could possibly be correlated. The classification inside distinct groups is nevertheless useful as these features rely on different models or mathematical concepts and therefore lead to interpretations of different nature. For instance, some stochastic features which are linked to diffusion phenomena could be positively correlated with positional features that also measure dispersion aspects of the signal, however in the first case the computation of the feature relies on a model of stochastic diffusion, whereas in the second case the dynamic of the trajectory is not taken into account leading to different interpretations.

### 2.4 Data collection

The clinical Research Ethics Committee approved the clinical study, registered at ANSM (ID RCB 2014‐A00222‐45).

#### 2.4.1 Participants

Elderly people with or without balance impairments were recruited during routine consultations in neurology departments (Val‐de‐Grace Hospital) and physical medicine and rehabilitation departments (Fernand Widal Hospital, Paris, France). In total, 133 individuals were included, 32 of them with recent history of falls (fallers: at least one fall in the previous 6 months). The participants included in this study were aged at least 60 years old.

#### 2.4.2 Experimental procedure

During these consultations and before the experiment, patients were asked about their history of falls in the last 6 months. Measurement of the COP displacement characteristics of the individuals was then performed using a Wii Balance Board (WBB; Nintendo), an alternative to laboratory grade force platform that have received increased attention in the recent years for quantifying postural control (Park & Lee, 2014; Severini et al., 2017). The use of the WBB is justified by its advantages in terms of convenience compared to laboratory force platforms. Its within‐device and between‐device reliability have been considered good and suitable for clinical settings (Clark et al., 2010), especially when preprocessing methods are applied to improve accuracy (Audiffren & Contal, 2016; Leach et al., 2014). During the static balance recording, patients were invited to stand on the platform. Data were collected by a custom software on a Samsung tablet (Android operating system version 2.0, Samsung), using Bluetooth L2CAP protocol. The balance test was performed two times with different conditions. First, the individuals stood in quiet stance, eyes open, looking straight ahead, arms at their sides, and feet comfortably positioned within the space provided on the WBB. After 10 s in this position, the trajectory of the COP was recorded for 25 s, a duration that has been shown to be sufficient to quantify postural control with variables (Bargiotas et al., 2018). Then the individuals were asked to close their eyes. After a further 10 s, the closed‐eye recording was started for 25 s. Between the two phases, there was no rest period, except in case of vertigo expressed by the subject. For the calculation of the variables, only the open‐eyes record is kept (a single repetition).

#### 2.4.3 Data preprocessing

Data preprocessing and analysis software were written using Python (v3.7, Python Software Foundation). The signals collected from the force platform were resampled at 25 Hz using SWARII (Audiffren & Contal, 2016), as the WBB is known to produce data at nonuniform frequency. Then, resulting force platform data were processed with a fourth‐order, zero‐lag, low‐pass Butterworth filter with a 10 Hz cutoff frequency in accordance to Hernandez et al. (2015).

Finally, due to the variability of foot positioning on the force platform, we chose to center the COP trajectories with respect to their arithmetic mean in our definitions and analysis, in line with most of previous studies (Prieto et al., 1996; Qiu & Xiong, 2015).

#### 2.4.4 Public dataset of human balance

Due to the lack of consensus on the methods of recording and analyzing posturographic signals, a public dataset was made available to allow comparison and testing of analysis methods (Santos & Duarte, 2016a). The dataset was constructed by a single experimenter at the Laboratory of Biomechanics and Motor Control at the Federal University of ABC, Brazil. Only the COP displacements of participants aged 60 and over, from this public dataset, were used to calculate the variables presented above.

The data in this set are resting balance recordings on a force platform (OPT400600‐1000; AMTI), for 60 s, at a sampling rate of 100 Hz. We use the averaged value on the three recordings made for each participant. To be consistent with our recording protocol, only data from the firm surface open‐eye recordings were used. Participants were asked to remain as steady as possible with their arms at their sides and to look at a target in front of them. The position of the feet was standardized as follows “with an angle of 20 degrees between them and their heels were kept 10 cm apart.” The force plate data were preprocessed through a 10 Hz fourth‐order, zero‐lag, low‐pass Butterworth filter. More details are available in the original publication (Santos & Duarte, 2016b).

#### 2.4.5 Sample characteristics

In total, 133 people recorded with the Wii Balance Board were included in this study. The demographics characteristics of participants are shown in Table 1. The mean age in this sample is high but corresponds to the populations presented by other authors (Aufauvre et al., 2005; Bauer et al., 2016a; Bigelow & Berme, 2011; Borg & Laxåback, 2010; Hewson et al., 2010; Maki et al., 1994; Muir et al., 2013; Ramdani et al., 2013). The incidence of the number of falls among people over 80 years of age was measured at nearly six falls per year (5,930 for women and 5,467 for men in 2009; Korhonen et al., 2012), which is consistent with the number of falls over the last 6 months in this study. In addition, the elderly participants’ characteristics of the public data base are presented in Table 1. We can note that the proportion of fallers in the two groups is close to 25%, although the average ages, the retrospective period during which falls are investigated and the average number of falls are different.

**TABLE 1:** Characteristics of study participants

	WBB dataset	Public dataset
Total	133	76
Men	72	16
Women	61	60
Age	78.7 (±6.7)	71.3 (±6.5)
BMI	24.4 (±4.1)	25.5 (±2.9)
Fallers	32 (6 last months)	19 (12 last months)
Number of falls (for fallers)	2.3 (±2.4)	3.8 (±11.7)

### 2.5 Descriptive analysis

#### 2.5.1 Variables distributions

In order to provide indicative values for the variables presented, we report the means and standard deviations for both populations, for each of the COP variables during eyes‐open recordings. Fallers and non‐fallers are aggregated for each database since the objective is not to discriminate between sub‐populations of the samples according to their fall risk or pathologies. Note that we chose not to include in our analysis two aforementioned variables, MEAN VALUE and VFY, in line with previous studies concerns about the considerable measurement errors that these features are prone to (Duarte & Freitas, 2010)—a problem that is compounded here as our study was multi‐centric, which inherently increased the probability of small variations between the participant feet position.

### 2.6 Open‐access code

A code enabling the calculation of all the COP variables that are presented is available at https://github.com/Jythen/code_descriptors_postural_control, as well as an oline demo on the IPOL website: https://ipolcore.ipol.im/demo/clientApp/demo.html?id=77777000137&key=C2AE7495B4E728249E4CE1905DA15186

## 3 RESULTS

### 3.1 General notations

In the following, we assume that the recorded COP trajectory contains N data points, sampled at constant frequency F_s_. T = N/F_s_ denotes the total duration of the signal in seconds (Table 2). For each 1 ≤ *n* ≤ *N*, *ML* *_n_*, (respectively *AP* *_n_*) denotes the coordinate of the COP position at time *n*/F_s_ on the ML axis, from left to right, (respectively the AP axis, from backward to forward). Then for each 1 ≤ *n* ≤ N

$$X_{n}={\text{ML}}_{n}‐\frac{1}{N}\sum_{i=1}^{N}{\text{ML}}_{i}$$

and

$$Y_{n}={\text{AP}}_{n}‐\frac{1}{N}\sum_{i=1}^{N}{\text{AP}}_{i}$$

represent the coordinates of the centered trajectories on the ML axis and AP axis, respectively. We also introduce the Radius signal (*R_n_*)_1≤_ *_n_*_≤__N_ as the Euclidean distance of the centered COP to the origin: for each 1 ≤ *n* ≤ N,

$$R_{n}=\sqrt{X_{n}^{2}+Y_{n}^{2}}$$

Finally, we define the covariance between the AP and ML variations of the COP as

$$\text{COV}=\frac{1}{N}\sum_{i=1}^{N}X_{n}Y_{n}$$

**TABLE 2:** General notations and signal transformations used in the definition of the features. For each quantity, we report the symbol used in this manuscript, the name of the symbol, the formula, the units, as well as the section where the feature is defined. Note that *S* is a placeholder symbol that can be replaced by both *X* (ML coordinates) and *Y* (AP coordinates)

Symbol	Name	Formula	Units	Section
T	Total duration of the signal	—	s	3.1
N	Number of points of the signal	—	—	
F_s_	Sampling frequency	N*/*T	Hz	
*ML* *_n_*	Mediolateral (ML) coordinates	—	cm	
*AP* *_n_*	Anteroposterior (AP) coordinates	—	cm	
*X_n_*	Centered ML coordinates	${\text{ML}}_{n}‐\frac{1}{N}\sum_{i=1}^{N}{\text{ML}}_{i}$	cm	
*Y_n_*	Centered AP coordinates	${\text{AP}}_{n}‐\frac{1}{N}\sum_{i=1}^{N}{\text{AP}}_{i}$	cm	
*R_n_*	Radius	$\sqrt{X_{n}^{2}+Y_{n}^{2}}$		
COV	Covariance AP	$\frac{1}{N}\sum_{i=1}^{N}X_{n}Y_{n}$	cm²	
*SD* *_n_*	Sway density	see Definition 1	s	3.3
*z_ℓ_*	Zero‐crossing	see Definition 3		
*p_ℓ_*	Peaks	see Definitions 4 and 2		
$V_{n}^{x}$	ML velocity	see Computing velocity and Notation	cm.s^‐1^	
$V_{n}^{y}$	AP velocity	see Computing velocity and Notation	cm.^‐1^	
$V_{n}$	Velocity norm	$\sqrt{{\left(V_{n}^{x}\right)}^{2}+{\left(V_{n}^{y}\right)}^{2}}$	cm.s^‐1^	
$\Gamma_{k}^{S}$	PSD of S for frequency *kFs*/*N*	—	cm^2^.Hz^‐1^	3.4
$M_{l}^{S}$	*ℓ*‐th spectral moment of *S*	$\sum_{k}f_{k}^{l}\Gamma_{k}^{s}$	cm^2^.Hz^−1^	
*MSD* ^S^ (Δ*t*)	Mean square displacement *S*	$\frac{\sum_{n}{\left(S_{n+F_{s}\Delta t}‐S_{n}\right)}^{2}}{N‐F_{s}\Delta_{t}}$	cm²	3.5

### 3.2 Positional variables

Variables are classified in this category if they depend on the COP positions and do not require the knowledge of its local displacements. Therefore, these descriptors can capture characteristics of the dispersion of the trajectory or a favored position for the point of support of the feet, and do not embed dynamic aspects of the signal, as they ignore the temporal nature of the data (Table 3).

#### 3.2.1 Mean value

The mean position, computed as the arithmetic average of the COP trajectory before centering, has been considered by Aufauvre et al. (2005), Stel et al. (2003), Brauer et al. (2000) and Maki et al. (1994), for the ML and AP coordinates. Importantly, previous works have disagreed with the use of this variable (Duarte & Freitas, 2010), given the variability in the placement of the feet on the force platform.

$$\text{MEAN}\;\text{ML}\;\frac{1}{N}\sum_{n=1}^{N}{\text{ML}}_{n}$$

$$\text{MEAN}\;\text{AP}\;\frac{1}{N}\sum_{n=1}^{N}{\text{AP}}_{n}$$

**TABLE 3:** Summary of the definition of the positional features. All the listed ML features can also be computed for the AP axis. For units, cm stands for centimeter, ° for degree (angle), and − for unitless

Feature	Full name	Formula	Units
MEAN ML	Mean ML coordinate	$\frac{1}{N}\Sigma_{n}{\text{ML}}_{n}$	cm
MEAN DIST. ML	Mean distance ML	$\frac{1}{N}\sum_{n}\left|X_{n}\right|$	cm
MEAN DIST.	Mean distance	$\frac{1}{N}\sum_{n}\left|R_{n}\right|$	cm
MAX ML	Maximal distance ML	max*_n_* |*X_n_* |	cm
MAX RADIUS	Maximal distance	max*_n_* |*R_n_* |	cm
RMS ML	Root mean square ML	$\sqrt{\frac{1}{N}\sum_{n}X_{n}^{2}}$	cm
RMS RADIUS	Root mean square radius	$\sqrt{\frac{1}{N}\sum_{n}R_{n}^{2}}$	cm
RANGE ML	Amplitude ML	max_n,m_ |*X_n_* − *X_m_* |	cm
RANGE ML‐AP	Amplitude ML‐AP	${\text{max}}_{1\leq n\leq m\leq N}\sqrt{{\left(X_{n}‐X_{m}\right)}^{2}+{\left(Y_{n}‐Y_{m}\right)}^{2}}$	cm
RANGE RATIO	Ratio of amplitudes	$\frac{\text{Range}\;\text{ML}}{\text{Range}\;\text{AP}}$	—
PLANAR DEV.	Planar deviation	$\sqrt{\text{RMS}\;{\text{ML}}^{2}+\text{RMS}\;{\text{AP}}^{2}}$	cm
COEF. SWAY DIR.	Coefficient of sway direction	$\frac{\text{COV}}{\text{RMS}\;\text{ML}\;\times \;\text{RMS}\;\text{AP}}$	—
95% CONF. AREA	95% confidence ellipse area	See Def.	cm²
PRINCIPAL SWAY DIR.	Principal sway direction	$\text{arccos}\left(\frac{\left|v_{2}\right|}{\sqrt{v_{1}^{2}+v_{2}^{2}}}\right)\times \frac{180}{\pi }$	°

#### 3.2.2 Mean distance

This feature represents the mean distance of the COP from the center of the trajectory (Maranesi et al., 2016; Prieto et al., 1996; Qiu & Xiong, 2015), which we estimate as the empirical average of the signal. Therefore, we define the mean distance using the centered signal, see the paragraph general notations. According to the authors, this descriptor could be related to the stability of the postural system. Age differences were found with higher values in the ML direction, especially in older women compared to younger participants or men (Kim et al., 2010). This variable also showed sensitivity to the size of the support base as it was found to decrease monotonically, especially in the ML direction, as the distance between the feet increased (Kim, Kwon, Jeon, Bang, et al., 2014). Regarding falls, Maranesi et al. (2016) have not either found significant differences for this feature between elderly fallers and non‐fallers in both ML and AP directions.

$$\text{MEAN}\;\text{DIST}.\;\text{ML}\;\frac{1}{N}\sum_{n=1}^{N}\left|X_{n}\right|$$

$$\text{MEAN}\;\text{DIST}.\;\text{AP}\;\frac{1}{N}\sum_{n=1}^{N}\left|Y_{n}\right|$$

$$\text{MEAN}\;\text{DIST}.\;\frac{1}{N}\sum_{n=1}^{N}\left|R_{n}\right|$$

#### 3.2.3 Maximal distance

This feature has been defined as the maximal distance of the COP from the centroid (Muir et al., 2013), which we interpret as the center of the trajectory. Similar to the mean distance, we define this feature as the maximum of the centered signal. This descriptor has been shown to be significantly greater in elderly fallers than in non‐fallers (Muir et al., 2013).

$$\text{MAX}\;\text{ML}\;{\text{max}}_{1\leq n\leq N}\left|X_{n}\right|$$

$$\text{MAX}\;\text{AP}\;{\text{max}}_{1\leq n\leq N}\left|Y_{n}\right|$$

$$\text{MAX}\;\text{RADIUS}\;{\text{max}}_{1\leq n\leq N}\;R_{n}$$

#### 3.2.4 Root mean square

The root mean square (RMS) is calculated on the centered trajectory. In the ML axis and AP axis it corresponds to the standard deviation of the trajectory and on the two‐dimensional signal it is the square root of the arithmetic mean of the squared radius (Prieto et al., 1996). Previous works have found changes associated with aging in this feature direction (Maki et al., 1994), particularly in the ML direction (Piirtola & Era, 2006; Swanenburg et al., 2010). (Bargiotas et al., 2018) also used successfully the RMS on the ML axis for their classification model between elderly fallers and elderly non‐fallers. However, Laughton et al. (2003) found significant differences between elderly non‐fallers and young participants for the AP standard deviation but not in the ML direction.

$$\text{RMS}\;\text{ML}\;\sqrt{\frac{1}{N}\sum_{n=1}^{N}X_{n}^{2}}$$

$$\text{RMS}\;\text{AP}\;\sqrt{\frac{1}{N}\sum_{n=1}^{N}Y_{n}^{2}}$$

$$\text{RMS}\;\text{RADIUS}\;\sqrt{\frac{1}{N}\sum_{n=1}^{N}R_{n}^{2}}$$

#### 3.2.5 Range (Amplitude)

The range, also called amplitude, of the COP path, has been widely used in the literature (Aufauvre et al., 2005; Bauer et al., 2010; 2016a; Howcroft et al., 2015, 2017; Laughton et al., 2003; Maranesi et al., 2016; Ramdani et al., 2013). In Prieto et al. (1996), the authors define the range as the maximal distance over two points of the stabilogram. Along one particular axis, this is mathematically equivalent to the distance between the maximum and the minimum positions of the signal. Previous works have shown contradictory results regarding the predictive power of this variable for the assessment of fall risks, but it has been shown that the RANGE in the ML direction differs between fallers and non‐fallers based on a meta‐analysis of data from elderly participants with a history of falls, in a previous systematic review (Quijoux et al., 2020).

$$\text{RANGE}\;\text{ML}\;{\text{max}}_{1\leq n\leq m\leq N}\left|X_{n}‐X_{m}\right|$$

$$\text{RANGE}\;\text{AP}\;{\text{max}}_{1\leq n\leq m\leq N}\left|Y_{n}‐Y_{m}\right|$$

$$\text{RANGE}\;\text{AP - ML}\;{\text{max}}_{1\leq n\leq m\leq N}\sqrt{{\left(X_{n}‐X_{m}\right)}^{2}+{\left(Y_{n}‐Y_{m}\right)}^{2}}$$

#### 3.2.6 Ratio of amplitudes (Quotient of both directions)

The ratio of the COP dynamics in ML and AP directions has been frequently studied in regards to the balance strategy involved to maintain erect posture in elderly people. Błaszczyk et al. (2014) computed the directional index as the ratio of the AP or ML path length divided by the total COP length. In Bauer et al. (2016a), the quotient of both directions is defined as the ratio of mediolateral amplitude over the anteroposterior amplitude, and this measure is shown to be significantly different between fallers and non‐fallers during eyes‐closed recordings (Bauer et al., 2010).

$$\text{RANGE}\;\text{RATIO}\;\frac{\text{RANGE}\;\text{ML}}{\text{RANGE}\;\text{AP}}$$

#### 3.2.7 Planar deviation

The planar deviation was defined by (Raymakers et al., 2005) as the square root of the sum of the variances of displacements in ML and AP directions. While it has been argued that this variable may be less discriminant than the range or the mean velocity (Raymakers et al., 2005) and has shown a small relative reliability, with an intraclass correlation coefficient (ICC) of 0.5, in eyes‐open condition, and a poor absolute reliability (Qiu & Xiong, 2015), the planar deviation has been used in multiple previous works to quantify human stability (Ilett et al., 2016; Xiong & Karim, 2013).

$$\text{PLANAR}\;\text{DEV}.\;\sqrt{\text{RMS}\;{\text{ML}}^{2}+\text{RMS}\;{\text{AP}}^{2}}$$

#### 3.2.8 Coefficient of sway direction

Bauer et al. (2016a) have defined the coefficient of sway direction as the ratio of the covariance between AP and ML directions over the marginal standard deviations, that is, as the coefficient of correlation between the ML and AP trajectories. This descriptor has been shown to be significantly associated with falls (Bauer et al., 2016a) in community‐dwelling older adults.

$$\text{COEF}.\;\text{SWAY}\;\text{DIR}.\;\frac{\text{COV}}{\text{RMS}\;\text{ML}\times \text{RMS}\;\text{AP}}$$

#### 3.2.9 95% confidence ellipse area (Sway area)

The confidence ellipse area (also called sway area) is defined as the area of the ellipse which contains the true mean of (*X_n_*, *Y_n_*)_1≤_ *_n_*_≤N_ with a probability of 95% (Schubert & Kirchner, 2014). An increase in this feature value among elderly people has been associated with a significantly higher risk of fall (Merlo et al., 2012). The confidence ellipse is derived from using the central limit theorem (Duarte & Freitas, 2010; Prieto et al., 1996; Schubert & Kirchner, 2014), which requires the assumption that the serie samples are independent and identically distributed. Let *F*_0.95,2,_ *_n_*_−2_ denote the 0.95‐quantile of the Fisher distribution with 2 and *n *− 2 degrees of freedom. Note that the unbiased versions of the covariance matrix could also be used (Schubert & Kirchner, 2014). The confidence ellipse can be approximated by the following formula:

$$95\%\;\text{CONF}.\;\text{AREA}\;2\pi \times \frac{N‐1}{N‐2}\times F_{0.95,2,N‐2}\times \sqrt{\text{RMS}\;{\text{ML}}^{2}\times \text{RMS}\;{\text{AP}}^{2}‐{\text{COV}}^{2}}$$

**FIGURE 1:**
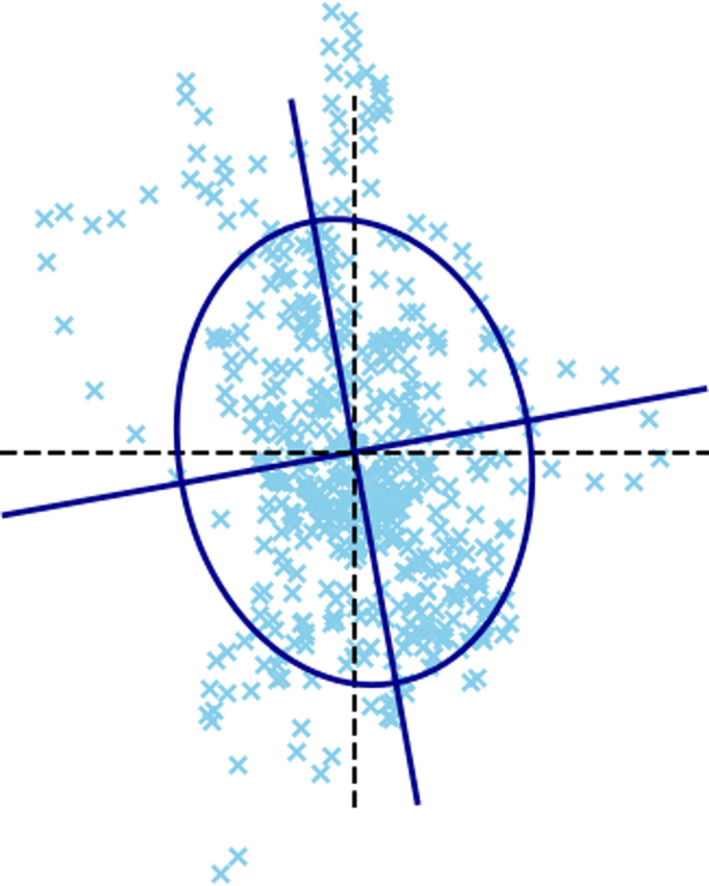
Illustration of the calculation of the 95% confidence ellipse. The feature is equal to the area of the ellipse

An illustration of the calculation of this feature is shown in Figure 1.

#### 3.2.10 Principal sway direction

Oliveira et al. (1996) introduced the principal sway direction as a tool to represent the relative contribution of the ML and AP components to the oscillations of the COP. The computation of the sway direction is based on a principal component analysis (PCA) which derives the direction of maximum dispersion of the COP trajectory. The principal direction is defined as the angle between 0° and 90°, between the AP axis and the direction of the main eigenvector produced by the PCA. Rocchi et al. (2004) have claimed that this variable provides a significant additional information regarding the COP dynamic, relative to other features. Let *v *= (*v*_1_, *v*_2_) denote the eigenvector associated with the highest variance produced by a PCA of the COP bi‐dimensional signal (*X_n_*, *Y_n_*)_1≤_ *_n_*_≤N_. Then the principal sway direction is defined as:

$$\text{SWAY}\;\text{DIRECTION}\;\arccos\left(\frac{\left|v_{2}\right|}{\sqrt{v_{1}^{2}+v_{2}^{2}}}\right)\times \frac{180}{\pi }$$

**FIGURE 2:**
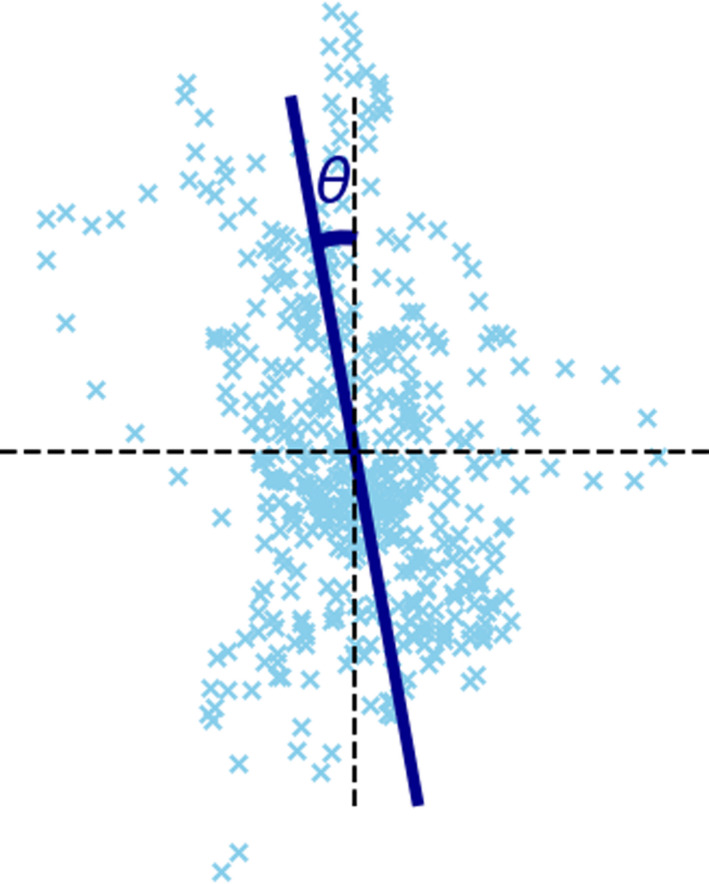
Illustration of the calculation of the principal sway direction. The feature is equal to the angle *θ*

An illustration of the calculation of this feature is shown in Figure 2.

### 3.3 Dynamic variables

These descriptors are based on the local displacements of the COP trajectory (Table 4). Most of them revolve around the quantification of the velocity of the signal, and consequently, are sensitive to additive noise, such as electromagnetic noise, and variation of the sampling frequency (see e.g. Press & Teukolsky, 1990; Schubert et al., 2012). Another quantity of interest for dynamic variables is the sway density, which is designed to encode the local stability of the COP signal. This is quantified by measuring around each point, the number of consecutive points which lie in a circle of a certain radius. This count is then divided by the sampling frequency. In this study we choose to use a radius of 3 mm, as it has been shown that the choice of the radius is not critical and that a value between 3 and 5 mm is adequate for most applications (Jacono et al., 2004).

**TABLE 4:** Summary of the definition of the dynamic features. All the listed ML features can also be computed for the AP axis. For units, cm stands for centimeter, s for seconds, Hz for Hertz, and − for unitless. *: This feature is obtained by summing non‐homogeneous term, and therefore has no valid units

Feature	Full name	Formula	Units
SWAY LENGTH ML	Sway length ML	$\sum_{n}\left|X_{n+1}‐X_{n}\right|$	
SWAY LENGTH	Total sway length	$\sum_{n}\sqrt{{\left(X_{n+1}‐X_{n}\right)}^{2}+{\left(Y_{n+1}‐Y_{n}\right)}^{2}}$	cm
MEAN SPD ML	Average velocity ML	SWAY LENGTH ML/T	cm.s^‐1^
MEAN SPD	Average velocity	SWAY LENGTH/T	cm.s^‐1^
AREA PER SEC.	Sway area per sec.	$\frac{1}{2T}\sum_{n}\left|X_{n+1}Y_{n}‐X_{n}Y_{n+1}\right|$	cm².s^‐1^
STD SPD ML.	Deviation velocity ML	$\sqrt{\frac{1}{N}\sum_{n}{\left(V_{n}^{x}‐\bar{V^{x}}\right)}^{2}}$	cm.s^‐1^
STD SPD.	Deviation velocity	$\sqrt{\frac{1}{N}\sum_{n}{\left(V_{n}‐\bar{V}\right)}^{2}}$	cm.s^‐1^
PHASE PLANE ML	ML phase plane parameter	$\sqrt{\text{RMS}\;{\text{ML}}^{2}+\text{STD}\;\text{SPD}\;{\text{ML}}^{2}}$	*
VFY	—	$\text{STD}\;{\text{SPD}}^{2}/\text{MEAN}\;\text{AP}$	cm.s^‐^^2^
LFS	Length over area	$\frac{\text{SWAY}\;\text{LENGTH}}{95\%\;\text{CONF}.\;\text{AREA}}$	cm
FRACTAL DIM	Fractal dimension	See Def. Fractal Dimension	—
SET OF ZERO CROSS. ML	Set of zero‐crossings ML	$Z^{V^{x}}$	
ZERO CROSS. ML	Number of zero‐crossings ML	$\#Z^{V^{x}}$	
PEAK VEL. + ML	Mean positive peak of ML Vel.	see Def.	cm.s^‐1^
PEAK VEL. ‐ ML	Mean negative peak of ML Vel.	see Def.	cm.s^‐1^
PEAK VEL. ML	Mean peak of ML velocity	$\frac{1}{K}\Sigma_{\ell }p_{\ell }^{V^{x}}$	cm.s^‐1^
PEAK SD	Mean peak of sway density	$\frac{1}{K}\Sigma_{\ell }p_{\ell }^{\text{SD}}$	s
DIST. PEAK SD	Mean spatial dist. between S.D. peaks	See Def	cm
MEAN FREQ. ML	Mean frequency ML	$\frac{1}{4\sqrt{2}}\times \frac{\text{MEAN}\;\text{SPD}\;\text{ML}}{\text{MEAN}\;\text{DIST}\;\text{ML}}$	Hz
MEAN FREQ. ML‐AP	Mean frequency	$\frac{1}{4\sqrt{2}}\times \frac{\text{MEAN}\;\text{SPD}}{\text{MEAN}\;\text{DIST}}$	Hz

Definition 1 (Sway density). The sway density at time n∆_t_ is defined as.

$${\text{SD}}_{n}=\frac{{\text{SD}}_{n}^{\left(+\right)}+{\text{SD}}_{n}^{\left(‐\right)}}{F_{s}}$$

where

$${\text{SD}}_{n}^{\left(+\right)}=\max\left\{q\geq 0,\;\forall p\leq q,\;\sqrt{{\left(X_{n+p}‐X_{n}\right)}^{2}+{\left(Y_{n+p}‐Y_{n}\right)}^{2}}\leq 3\;\text{mm}\right\}$$

$${\text{SD}}_{n}^{\left(‐\right)}=\max\left\{q\geq 0,\;\forall p\leq q,\;\sqrt{{\left(X_{n‐p}‐X_{n}\right)}^{2}+{\left(Y_{n‐p}‐Y_{n}\right)}^{2}}\leq 3\;\text{mm}\right\}$$

Definition 2 (Peaks of sway density). To compute the peaks of the sway density, the signal is first low‐pass filtered at 2.5 Hz with a Butterworth filter of order 4 (Jacono et al., 2004). Let ${\tilde{\mathrm{SD}}}_{n}$ represent the sway density signal obtained after filtering. Then, the peaks of SD_n_ are defined as the local maximum of the filtered signal that is, they occur at the indices in {$n_{p_{1}}^{S},\;\ldots ,\;n_{p_{k}}^{S}$} such that for all $k\in \left\{1,...,\;K\right\},\;1<n_{p_{k}}^{S}<N,\;{\tilde{\text{SD}}}_{n_{p_{k}}^{S}}>\;{\tilde{\text{SD}}}_{n_{p_{k}}^{S}‐1}$ and ${\tilde{\text{SD}}}_{n_{p_{k}}^{S}}>{\tilde{\text{SD}}}_{n_{p_{k}}^{S}}+1$.

**FIGURE 3:**
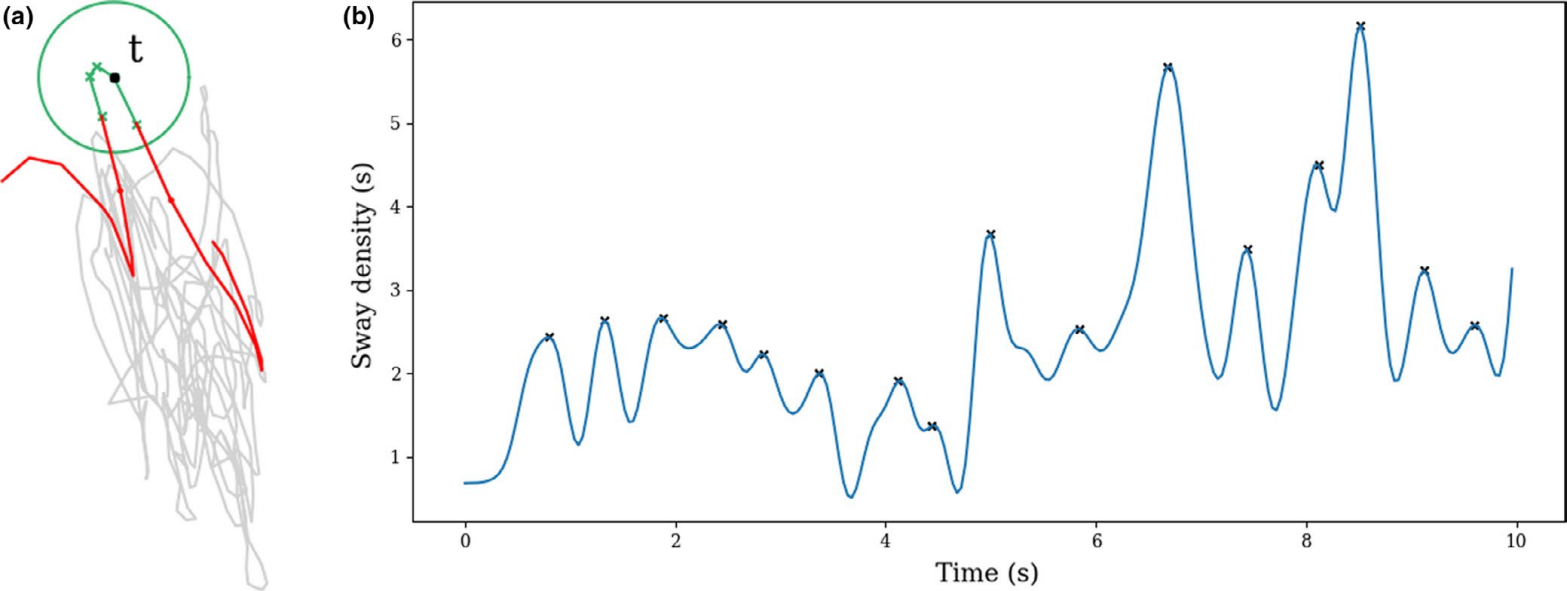
Illustration of the sway density computation and the peaks computation. (a) Illustration of the computation of the sway density at time *t*. In this example, four consecutive points fall in the circle of radius 3mm, therefore the sway density at time *t* is equal to 4/ F_s_. (b) Example of filtered trajectory of the sway density over time. The black crosses indicate the position of peaks identified using Definition 2

An example of peaks identified on a sway density signal is shown in Figure 3b.

#### 3.3.1 Computing velocity

The COP trajectory recorded using force platforms is by nature a noisy signal. To address this problem, common preprocessing methods, such as low‐pass filters are used to remove the high‐frequency components of the noise. However, there is no consensus on the frequency threshold that separates body sway from sensor noise. For instance, values of 5, 10, and 20 Hz have been proposed by Geurts et al. (1993), Hernandez et al. (2015) and Huurnink et al. (2013). This choice has a significant impact on the computation of the COP velocity, in particular when using discrete derivative formula. Therefore, and to limit the influence of the hyperparameters and the force platform characteristics, it is important to use robust methods such as spline interpolation or Savitzky–Golay filters to differentiate the signal (Curtain & Pritchard, 1977; Press & Teukolsky, 1990; Savitzky & Golay, 1964).

**Notation** In the following, *V^x^* =  (*V_n_^x^*)_1≤_ *_n_*_≤__N_ and *V^y^* = (*V_n_^y^*)_1≤_ *_n_*_≤__N_ represent the estimations of the COP velocities in the ML axis and AP axis, respectively. In our experiments, they are computed using a Savitsky–Golay filter with a polynomial of order 3 and a filter window of length 5. *V* represents the norm of the velocity, that is, for each 1 ≤ *n* ≤ N,

$$V_{n}=\sqrt{{\left(V_{n}^{x}\right)}^{2}+{\left(V_{n}^{y}\right)}^{2}}$$

The mean values of $V^{x}$, $V^{y}$, and V are, respectively, denoted by $\bar{V^{x}}$, $\bar{V^{y}},$ and $\bar{V}.$

Definition 3 (Zero‐crossing points of velocity) Let $V={\left(V_{n}\right)}_{1\leq n\leq N}$ stand for the velocity signal in the ML axis or AP axis. The zero‐crossing points *z*_1_, …, *z_J_*, are the variables in {1, …, N} verifying the following conditions:

1. For all $\ell \in \left\{1,\;\ldots ,\;J\right\},\;V_{z_{\ell ‐1}}\times V_{z_{\ell }}\leq 0$ and $V_{z_{\ell }}\neq 0$
2. $V_{z_{1}}\times V_{n_{0}}<0$ and for all $\ell \in \left\{2,\;\ldots ,\;J\right\},\;V_{z_{\ell }}\times V_{z_{\ell }‐1}<0$

**FIGURE 4:**
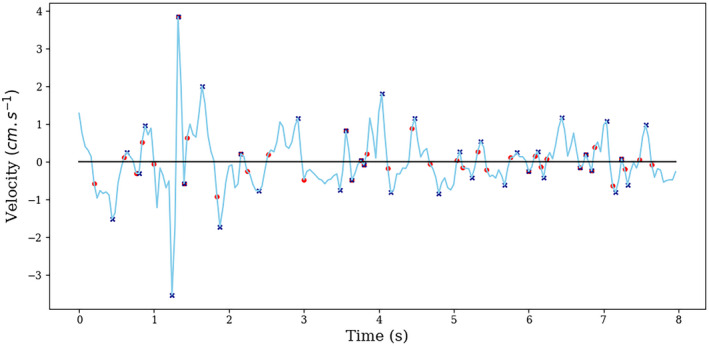
An example of velocity signal. The red dots indicate zero‐crossings identified using Definition 3 and the black crosses indicate the position of peaks identified using Definition 4

Definition 4 (Peaks of velocity) Let *V* = (*V_n_*)_1≤_ *_n_*_≤__N_ stand for *V^x^* or *V^y^*. Let *z*_1_, …, *z_J_* be the zero‐crossing points of *V*. Then for all 1 ≤ ℓ ≤ *K* = *J* − 1, the ℓ‐th peak of *V* occurs at the sampling variable $n_{p_{\ell }^{V}}$ and is equal to $p_{\ell }^{V}$, where $n_{p_{\ell }^{V}}=\arg\underset{}{{\text{max}}_{n\in \left\{1,\ldots ,N\right\},z_{\ell }\leq n\leq z_{\ell +1}‐1}}\left|V_{n}\right|$ and $p_{\ell }^{V}=V_{n_{p_{\ell }^{V}}}$. An example of peaks identified on a velocity signal is shown in Figure 4.

#### 3.3.2 Mean velocity (Normalized sway length, sway path)

The mean velocity of the COP is one of the most widely used variables. Overall, the mean velocity is considered as one of the most reliable feature, especially in the AP direction (Low et al., 2017). This variable has been shown to be influenced by age‐related postural alterations, under both eyes‐open and eyes‐closed conditions (Prieto et al., 1993, 1996) and to be predictive of the risk of falling (Howcroft et al., 2017). Indeed, the COP movement velocity was significantly correlated with age‐related neuromuscular phenomena such as loss of plantar flexor muscle volume (Kouzaki & Masani, 2012), tremors (Kouzaki & Masani, 2012), or an increase in the co‐contraction strategy of agonist and antagonist muscles of the leg (Benjuya et al., 2004; Carpenter, Frank, Silcher, et al., 2001; Ho & Bendrups, 2002; Nelson‐Wong et al., 2012). The perception of the COP movement velocity could be an important factor in the control of ankle extensor activity through anticipatory strategies (Masani, 2003; Sun et al., 2019), highlighting the impact of age‐related neuromuscular deterioration on static balance, with significant differences between eyes‐open or eyes‐closed condition (Howcroft et al., 2015) and more generally on the risk of falling (Brauer et al., 2000; Kwok et al., 2015). For a constant sampling interval, the mean velocity is defined as the sum of the distances between consecutive points, also called sway length, divided by the duration of the recording. Therefore, the mean velocity can be seen as a normalized version, with respect to the duration, of the sway length, which has been previously cited as the most common feature in the literature to evaluate the effect of exercise interventions (Low et al., 2017), and has been shown to distinguish people at risk of falling from healthy people (Kantner et al., 1991).

$$\text{SWAY}\;\text{LENGTH}\;\text{ML}\;\sum_{n=1}^{N‐1}\left|X_{n+1}‐X_{n}\right|$$

$$\text{SWAY}\;\text{LENGTH}\;\text{AP}\;\sum_{n=1}^{N‐1}\left|Y_{n+1}‐Y_{n}\right|$$

$$\text{SWAY}\;\text{LENGTH}\;\sum_{n=1}^{N‐1}\sqrt{{\left(X_{n+1}‐X_{n}\right)}^{2}+{\left(Y_{n+1}‐Y_{n}\right)}^{2}}$$

$$\text{MEAN}\;\text{SPD}\;\text{ML}\;\frac{\text{SWAY}\;\text{LENGTH}\;\text{ML}}{T}$$

$$\text{MEAN}\;\text{SPD}\;\text{AP}\;\frac{\text{SWAY}\;\text{LENGTH}\;\text{AP}}{T}$$

$$\text{MEAN}\;\text{SPD}\;\frac{\text{SWAY}\;\text{LENGTH}}{T}$$

Note that it is also possible to compute the mean velocity differently, using the Savitzky–Golay derivative previously discussed in the paragraph *Computing Velocity*. While not mathematically equivalent, these two definitions lead to similar values of mean velocity, as the average operator is robust to smooth interpolation such as Savitzky–Golay filters. Therefore we present here the normalized sway length formulation, which is frequently used in clinical studies (Low et al., 2017).

#### 3.3.3 Sway area per second

**FIGURE 5:**
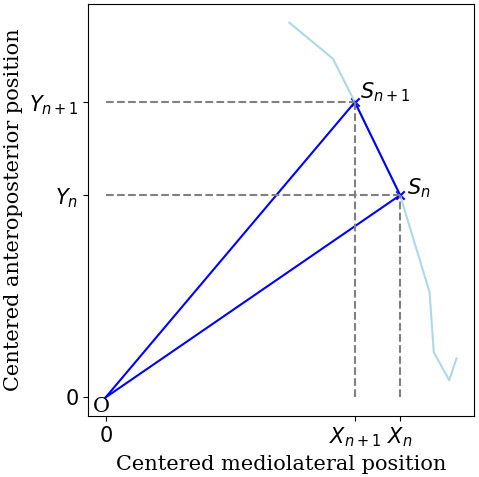
The sway area per second sums the area of the successive triangles *OS_n_S_n_*_+1_ (in blue) formed at each time *n* by the points of the signal and the center of the trajectory *O*

This variable evaluates the average area circumscribed by the COP for each 1 s time interval. The interval duration used for its calculation may vary between studies (Hufschmidt et al., 1980), and is not always clearly stated in the literature (Maranesi et al., 2016). The sway area per second is computed by adding the area of the triangles whose vertices are two consecutive points of the COP trajectory and the mean position of the COP (Hufschmidt et al., 1980; Prieto et al., 1996). Figure 5 shows an example of the triangle formed at a specific time for a real signal. This feature has been shown to significantly differ between non‐fallers and fallers (Lichtenstein et al., 1988; Maranesi et al., 2016; Pajala et al., 2008).

$$\text{AREA}\;\text{PER}\;\text{SEC}.\;\frac{1}{2T}\sum_{n=1}^{N‐1}\left|X_{n+1}Y_{n}‐X_{n}Y_{n+1}\right|$$

#### 3.3.4 Phase plane parameter

This feature is thought to express the dispersion of both the velocity and the position of the COP (Riley et al., 1995). It has been claimed that this variable provides insight into this dynamic aspect of balance control, and significantly differs between young healthy and elderly participants (Raymakers et al., 2005). Moreover, the phase plane parameter has been found to be reliable in both open‐eyes and closed‐eyes conditions (Moghadam et al., 2011; Qiu & Xiong, 2015). However it should be noted that the two terms that are added together, the standard deviation of position and the standard deviation of velocity, are not homogeneous.

$$\text{STD}\;\text{SPD}\;\text{ML}\;\sqrt{\frac{1}{N}\sum_{n=1}^{N}{\left(V_{n}^{x}‐\bar{V^{x}}\right)}^{2}}$$

$$\text{STD}\;\text{SPD}\;\text{AP}\;\sqrt{\frac{1}{N}\sum_{n=1}^{N}{\left(V_{n}^{y}‐\bar{V^{y}}\right)}^{2}}$$

$$\text{PHASE}\;\text{PLAN}\;\text{ML}\;\sqrt{\text{RMS}\;{\text{ML}}^{2}+\text{STD}\;\text{SPD}\;{\text{ML}}^{2}}$$

$$\text{PHASE}\;\text{PLAN}\;\text{AP}\;\sqrt{\text{RMS}\;{\text{AP}}^{2}+\text{STD}\;\text{SPD}\;{\text{AP}}^{2}}$$

#### 3.3.5 VFY

Gagey and Gentaz (1993) first^1^ defined this parameter as the variance of the COP velocity divided by the mean position of the COP on the AP axis, but this definition was contested by more recent work (Gagey, 1999). However, this definition is still commonly used (see e.g. Aufauvre et al., 2005), therefore we chose to report it below. The VFY could be correlated with the tension of the posterior leg muscles (due to both viscoelasticity and basic tone; de Tauzia et al., 2010; Gagey & Gentaz, 1993) but the link with physiology has yet to be demonstrated. Importantly, the VFY suffers from the same drawback as the mean value does, due to the variability in the placement of the feet on the force platform (Duarte & Freitas, 2010).

$$\text{STD}\;\text{SPD}\;\sqrt{\frac{1}{N}\sum_{n=1}^{N}{\left(V_{n}‐\bar{V}\right)}^{2}}$$

$${\text{VFY}}^{1}\;\frac{\text{STD}\;{\text{SPD}}^{2}}{\text{MEAN}\;\text{AP}}$$

#### 3.3.6 Length over area (LFS)

In Aufauvre et al. (2005), the length over area is defined as the total length of the sway path over the surface of the circumscribing area (circle or ellipse). In their study, the authors did not find any significant difference between fallers and non‐fallers for this variable, or according to whether the eyes were open or closed. Kim et al. (2019) have shown that the length over area was correlated in eyes‐closed condition with mild‐to‐moderate traumatic brain injury, showing a poorest balance control when the white matter trauma is more severe.

$$\text{LFS}\;\frac{\text{SWAY}\;\text{LENGTH}}{95\text{\%}\;\;\text{CONF}.\text{AREA}}$$

#### 3.3.7 Fractal dimension

The fractal dimension is a unitless measure of the degree to which a curve fills the space it is embedded in Prieto et al. (1996). Previous works have claimed that the fractal dimension of the COP is one of the most reliable sway variable for differentiating among age groups and pathologies (Myklebust et al., 1995). Three main methods are used to compute the fractal dimension (Prieto et al., 1996). In a first model, the area of the stabilogram is approximated using a circle including all the points of the COP trajectory, which generally over estimates the area enclosed by the signal (Prieto et al., 1996). In the two other methods, the area is computed using either a confidence circle or a confidence ellipse. We present hereafter the formula using the confidence ellipse, which is more flexible. The value of the fractal dimension could increase in healthy adults when the eyes are closed (Tassani et al., 2019) or when wearing orthopedic insoles (Bateni, 2013). Significantly higher values were found in young participants than in elderly people during eyes‐open recording (Qiu & Xiong, 2015). These findings are more in line with an improvement in stability as the value of the fractal dimension increases.

$$\text{FRACTAL}\;\text{DIM}\frac{\log N}{\log N+\log\sqrt{\frac{4}{\pi }\times 95\%\;\text{CONF}.\;\text{AREA}}‐\log\;\text{SWAY}\;\text{LENGTH}}$$

#### 3.3.8 Zero‐crossing (of velocity)

This variable is defined as the number of times that the COP velocity crosses the zero value axe (Jeong et al., 2007). Tuunainen et al. indicated that “zero‐crossing velocity showed a high rate of velocity change around the neutral position of stance” (Tuunainen et al., 2013). The latter found an association between this variable and falls, but no significant difference between fallers and non‐fallers, which is in line with previous results that found no significant difference even when comparing the two groups of older people to healthy subjects (Hewson et al., 2010). Let $Z^{V^{x}}$ and $Z^{V^{y}}$ denote the sets of zero‐crossing points of *V^x^* and *V^y^*, respectively, given by Definition 3. The zero‐crossing variables represent the number of zero‐crossing points in each direction:

$$\text{ZERO}\;\text{CROSS}.\;\text{ML}\;\#Z^{V^{x}}$$

$$\text{ZERO}\;\text{CROSS}.\;\text{AP}\;\#Z^{V^{y}}$$

#### 3.3.9 Mean velocity peak

A velocity peak has been defined as the maximal value between two zero‐crossing points (Hewson et al., 2010). The positive peaks of velocity, which correspond to displacements forward and to the right in the AP axis and ML axis, respectively, may be considered separately from negative peaks, which correspond to displacements backward and to the left in the AP axis and ML axis, respectively. The mean AP velocity peak has been shown to discriminate between elderly fallers and non‐fallers (Hewson et al., 2010). An increase in the absolute value would indicate poorest postural control. The zero‐crossing velocity variables are correlated with each other in each direction (*R* = 0.88) but may be more weakly correlated with other variables, especially with positional variables (*R* < 0.8), in the older population (Rasku et al., 2012). Peak COP velocity has also been previously correlated with the severity of knee osteoarthrosis during the transition task from double‐leg to single‐leg standing (Sabashi et al., 2021).

$$\mathrm{PEAK}\;\mathrm{AP}\;\mathrm{VEL}.+\mathrm{ML}\;\;\;\;\;\frac{\sum_{l=1}^{K}p_{l}^{V^{x}}\times 1_{\left\{p_{l}^{V^{x}}>0\right\}}}{\sum_{l=1}^{K}1_{\left\{p_{l}^{V^{x}}>0\right\}}}$$

$$\mathrm{PEAK}\;\mathrm{AP}\;\mathrm{VEL}.\;‐\mathrm{ML}\;\;\;\;\;\frac{\sum_{l=1}^{K}p_{l}^{V^{x}}\times l_{\left\{p_{l}^{V^{x}}<0\right\}}}{\sum_{l=1}^{K}l_{\left\{p_{l}^{V^{x}}<0\right\}}}$$

$$\mathrm{PEAK}\;\mathrm{AP}\;\mathrm{VEL}.\;\mathrm{ML}\;\;\;\;\;\frac{1}{K}\sum_{l=1}^{K}p_{l}^{V^{x}}$$

These variables are similarly defined in the AP axis by replacing *V^x^* by *V^y^*.

#### 3.3.10 Mean sway density peak

With the idea that postural control in quiet standing is governed by two major mechanisms (intrinsic feedback and anticipatory feedforward), previous studies have focused on structural posturographic parameters. (Baratto et al., 2002) have proposed a model in which these mechanisms, modulated by ankle muscle activation and the internal inverted pendulum model, respectively, distinguish between short‐ and long‐term factors. From this hypothesis, they propose to analyze the sway density (SD), counting the number of consecutive samples of the posturographic trajectory that, for each instant, fall within a circle of given radius defined by the operator (typically between 3 and 5 mm, Jacono et al., 2004). In the resulting signal, the SD peaks (high values of the number of points in the circle) correspond to the moments when the ankle torque and the associated motor control systems enable relatively stable COP displacements.

$$\text{PEAK}\;\text{SD}\;\frac{1}{K}\sum_{l=1}^{K}p_{l}^{\text{SD}}$$

#### 3.3.11 Mean spatial distance between sway density peaks

While peaks of SD correspond to relatively stable COP displacements, valleys (low values of SD) are interpreted as destabilization phases in which the ankle torque rapidly changes from one stable state to another, similar to a micro‐fall. Hence, the distance between two consecutive peaks in the SD represent a micro‐fall or a period of destabilization for (Baratto et al., 2002). This saccade could correspond to the amplitude of the posturographic command or “the amount of change in torque required for stabilization” (Vieira et al., 2009b). The values of the “jump” from one posturographic target to the next can be averaged to compute the mean spatial distance between peaks. The mean distance between peaks seems to increase significantly when the eyes are closed (Kim et al., 2012; Vieira et al., 2009b), in old age (Kim et al., 2012) or with history of past falls (Audiffren et al., 2016; Maranesi et al., 2016).

$$\text{DIST}.\;\text{PEAK}\;\text{SD}\;\frac{1}{K}\sum_{l=1}^{K}\sqrt{{\left(X_{\tau_{l+1}}‐X_{\tau_{l}}\right)}^{2}+{\left(Y_{\tau_{l+1}}‐Y_{\tau_{l}}\right)}^{2}}$$

#### 3.3.12 Mean frequency

The mean frequency is defined by Prieto et al. (1996) as the rotational frequency, considering the total length of the COP as a trajectory around a circle with a radius equals to the mean distance. This variable is proportional to the ratio of the mean velocity to the mean distance, which has been studied in Hufschmidt et al. (1980). In Maki et al. (1994), the mean frequency did distinguish fallers from non‐fallers in prospective follow‐up and provided limited information to discriminate fallers based on the history of falls in retrospective studies (König et al., 2014; Maranesi et al., 2016). However, it has been argued that MEAN FREQUENCY, especially in the AP direction, can be used to distinguish elderly fallers from non‐fallers (McGrath et al., 2012), and is reliable (Qiu & Xiong, 2015).

$$\text{MEAN}\;\text{FREQ}.\;\text{ML}\;\frac{1}{4\sqrt{2}}\times \frac{\text{MEAN}\;\text{SPD}\;\text{ML}}{\text{MEAN}\;\text{DIST}\;\text{ML}}$$

$$\text{MEAN}\;\text{FREQ}.\;\text{AP}\;\frac{1}{4\sqrt{2}}\times \frac{\text{MEAN}\;\text{SPD}\;\text{AP}}{\text{MEAN}\;\text{DIST}\;\text{AP}}$$

$$\text{MEAN}\;\text{FREQ}.\;\text{ML - AP}\;\frac{1}{2\pi }\times \frac{\text{MEAN}\;\text{SPD}}{\text{MEAN}\;\text{DIST}}$$

### 3.4 Frequency variables

This category is similar to the one presented in Prieto et al. (1996), and includes the variables used to describe the power spectral density of the COP trajectory. Similar to the dynamic variables, these descriptors are influenced by the sampling frequency of the force platform as well as the signal preprocessing (Table 5).

**TABLE 5:** Summary of the definitions of the frequency features. All the listed features can also be computed for the AP coordinates. For units, cm stands for centimeter, Hz for Hertz, and − for unitless

Feature	Full name	Formula	Units
total power ml	Total power ML	$\sum_{k=k_{\inf}}^{k_{\sup}}\Gamma_{k}^{X}$	cm^2^
50% POWER FREQ ML	Median of PSD ML	$\inf\left\{\left.k^{*}\in N,\;\sum_{k=k_{\inf}}^{k^{*}}\Gamma_{k}^{X}\geq 0.5\sum_{k=k_{\inf}}^{k_{\sup}}\Gamma_{k}^{X}\right\}\times \frac{F_{s}}{N}\right.$	Hz
95% POWER FREQ ML	95% percentile of PSD ML	$\inf\left\{\left.k^{*}\in N,\;\sum_{k=k_{\inf}}^{k^{*}}\Gamma_{k}^{X}\geq 0.95\sum_{k=k_{\inf}}^{k_{\sup}}\Gamma_{k}^{X}\right\}\times \frac{F_{s}}{N}\right.$	Hz
POWER MODE ML	Mode of PSD	$\frac{F_{s}}{N}\times \text{arg}\;{\text{max}}_{k_{\inf}\leq k\leq k_{\sup}}\Gamma_{k}^{X}$	Hz
CENTROIDAL FREQ ML	Centroidal frequency ML	$\sqrt{\frac{M_{2}^{X}}{M_{0}^{X}}}$	Hz
FREQ. DISP. ML	Frequency dispersion ML	$\sqrt{1‐\frac{{\left(M_{1}^{X}\right)}^{2}}{M_{2}^{X}M_{0}^{X}}}$	**—**
ENERGY ≤0.5 HZ ML	Energy content below 0.5 Hz ML	$\sum_{f_{\inf}\leq f_{k}\leq 0.5}\Gamma^{X}\left(f_{k}\right)$	cm²
ENERGY 0.5–2 HZ ML	Energy content 0.5–2 Hz ML	$\sum_{0.5\leq f_{k}\leq 2}\Gamma^{X}\left(f_{k}\right)$	cm²
ENERGY >2HZ ML	Energy content above 2 Hz ML	$\sum_{2<f_{k}\leq f_{\sup}}\Gamma^{X}\left(f_{k}\right)$	cm²
FREQ. QUOTIENT ML	Frequency quotient	$\frac{\sum_{2\leq f_{k}\leq 5}\Gamma^{X}\left(f_{k}\right)}{\sum_{f_{\inf}\leq f_{k}\leq 2}\Gamma^{X}\left(f_{k}\right)}$	—

**Notation** In the following, $\Gamma_{k}^{X}=\Gamma^{X}\left(f_{k}\right)$ denotes the power spectral density (PSD) coefficient of X corresponding to the frequency $f_{k}=k\frac{F_{s}}{N}$, for $k\in \left\{1,\;\ldots ,\;N/2\right\}$ if N is even, $k\in \left\{1,\;\ldots ,\;\left(N‐1\right)/2\right\}$ otherwise. The frequency‐domain measures are calculated for the frequency range from $f_{\inf}=0.15\;\text{Hz}$ to $f_{\sup}=5\;\text{Hz}$, which corresponds to variables $k_{\inf}=\left[0.15\frac{N}{F_{s}}\right]+1$ and $k_{\sup}=\left[5\frac{N}{F_{s}}\right]$, an interval likely to provide significant information about the postural control system (Prieto et al., 1996). We denote by

$$M_{l}^{X}=\sum_{k=k_{inf}}^{k_{sup}}f_{k}^{l}\Gamma_{k}^{X}$$

The *ℓ*‐th moment of the PSD. $\Gamma_{k}^{Y}$ and $M_{1}^{Y}$ are defined similarly. In our experiments, we estimate the PSD using Welch's method with 10‐s segments, with 50% overlapping and linear detrending (Vieira et al., 2009a).

#### 3.4.1 Total power

The total power is the energy contained in the entire power spectrum (Prieto et al., 1996). Previous works have shown that the TOTAL POWER may be significantly larger in elderly participants compared to young adults (Kim et al., 2010; Loughlin & Redfern, 2001). In both groups, TOTAL POWER seems to be positively correlated with height and also be dependent on the base‐of‐support in ML direction (Chiari et al., 2002, Kim, Kwon, Jeon, Eom, et al., 2014).

$$\text{TOTAL}\;\text{POWER}\;\text{ML}\;\sum_{k=k_{\inf}}^{k_{\sup}}\Gamma_{k}^{X}$$

The same feature is defined for the AP axis through replacing $\Gamma_{k}^{X}$ by $\Gamma_{k}^{Y}$.

Quantiles of PSD (Baratto et al., 2002) have shown that the frequency containing approximately 80% (from 70.7% to 95%) of the PSD may be of interest to the quantification of postural control. However these percentage values of interest vary significantly between studies. In (Maranesi et al., 2016) the authors proposed the values from 50 to 95%, which were in turn used in (Howcroft et al., 2017). In particular, the 50% power frequency has been shown to be sensitive to muscle fatigue (Corbeil et al., 2003).

$50\%\;\mathrm{POWER}\;\mathrm{FREQ}.\mathrm{ML}\;inf\left\{k⋆\in N,\sum_{k=k_{inf}}^{k⋆}\Gamma_{k}^{X}\geq 0.5\sum_{k=k_{inf}}^{k_{sup}}\Gamma_{k}^{X}\right\}\times \frac{F_{s}}{N}$

$95\%\;\mathrm{POWER}\;\mathrm{FREQ}.\mathrm{ML}\;inf\left\{k⋆\in N,\sum_{k=k_{inf}}^{k⋆}\Gamma_{k}^{X}\geq 0.95\sum_{k=k_{inf}}^{k_{sup}}\Gamma_{k}^{X}\right\}\times \frac{F_{s}}{N}$

The same features are defined for the AP axis through replacing $\Gamma_{k}^{X}$

#### 3.4.2 PSD mode

The power spectrum density mode is the dominant frequency of the PSD (McClenaghan et al., 1995). This variable has previously been used to track changes in the physiological rhythm, under the assumption that it would reflect modifications of the postural control strategy (Mackey & Glass, 1977; McClenaghan et al., 1995; Williams et al., 1997). This parameter showed no significant difference between fallers and non‐fallers in either the AP or ML direction (Lajoie, 2004).

$$\text{Power}\;\text{Model}\;\text{ML}\;\frac{F_{s}}{N}\times \arg\max_{k_{\inf}\leq k\leq k_{\sup}}\Gamma_{k}^{X}$$

The same features are defined for the AP axis through replacing $\Gamma_{k}^{X}$ by $\Gamma_{k}^{Y}$.

#### 3.4.3 Centroidal frequency and frequency dispersion

Both of these metrics measure the concentration of the spectral mass in the PSD. The centroidal frequency locates where the spectral mass is concentrated, and is defined as the square root of the ratio of the second to the zeroth spectral moments. The frequency dispersion is a measure of the variability in the frequency content of the power spectral density, ranging from zero (no dispersion) to one (uniform spectral bandwidth; Prieto et al., 1996; Vanmarcke, 1972). In previous studies, these two variables have been found not to be significantly different between young individuals and elderly (Loughlin & Redfern, 2001).

$$\text{CENTROIDAL}\;\text{FREQ}\;\text{ML}\;\sqrt{\frac{M_{2}^{X}}{M_{0}^{X}}}$$

$$\text{FREQ}.\;\text{DISP}.\;\text{ML}\;\sqrt{1‐\frac{{\left(M_{1}^{X}\right)}^{2}}{M_{2}^{X}M_{0}^{X}}}$$

The same features are defined for the AP axis through replacing $M_{l}^{X}$ by $M_{l}^{Y}$.

#### 3.4.4 Energy content of frequencies intervals

The energy contents of particular frequency bands have raised significant interest in the evaluation of postural control. In Soames and Atha (1982), the energy content (in the AP direction) of the intervals 0.3–0.45, 0.6–0.75, and 1.05–1.20 Hz was considered, while in the ML direction, the intervals were 0.30–0.45, 0.45–0.60, and 0.75–0.90 Hz. Since then, other studies have proposed less granular intervals, to focus on low frequencies (between 0 and 2 Hz) and high frequencies (2–5 Hz; Aufauvre et al., 2005; Bauer et al., 2010; 2016a). This difference is partly due to the population studied: while Soames and Atha (1982) have studied the balance of young healthy people the more recent studies were interested in older subjects. Similarly, Baloh et al. (1998) have proposed to study the quotient of the power of high frequencies (2–5 Hz) over the power of low frequencies (0–2 Hz). This quotient has been shown to significantly differ between young and elderly people (Baloh et al., 1994), and may be relevant to evaluate the influence of neurological impairment over postural control (Table 6; Sullivan et al., 2006, 2010, 2015).

$$\text{ENERGY}\leq 0.5\;\text{Hz}\;\sum_{f_{\inf}<f_{k}\leq 0.5}\Gamma^{X}\left(f_{k}\right)$$

$$\text{ENERGY}\;0.5‐2\;\text{Hz}\;\sum_{0.5<f_{k}\leq 2}\Gamma^{X}\left(f_{k}\right)$$

$$\text{ENERGY}\;>2\;\text{Hz}\;\sum_{2<f_{k}\leq f_{\sup}}\Gamma^{X}\left(f_{k}\right)$$

$$\text{FREQ}.\;\text{QUOTIENT}\;\frac{\sum_{2<f_{k}\leq 5}\Gamma^{X}\left(f_{k}\right)}{\sum_{f_{\inf}<f_{k}\leq 2}\Gamma^{X}\left(f_{k}\right)}$$

The same features are defined for the AP axis through replacing $\Gamma_{k}^{X}$ by $\Gamma_{k}^{Y}$.

**TABLE 6:** Summary of the definition of the stochastic features. All the listed features can also be computed for the AP coordinates. Units are not reported since they are undefined in the stochastic models

Feature	Full name	Formula
SHORT‐TERM DIFF. ML	Short‐term diffusion coefficient ML	$\exp\left({\hat{\alpha }}_{s}^{X}\right)$
LONG‐TERM DIFF. ML	Long‐term diffusion coefficient ML	$\exp\left({\hat{\alpha }}_{l}^{X}\right)$
SHORT‐TERM SCAL. ML	Short‐term scaling coefficient ML	${\hat{\beta }}_{s}^{X}/2$
LONG‐TERM SCAL. ML	Long‐term scaling coefficient ML	${\hat{\beta }}_{l}^{X}/2$
CRIT. TIME ML	Critical time ML	$\exp\left(\frac{{\hat{\beta }}_{s}^{X}‐{\hat{\beta }}_{l}^{X}}{{\hat{\alpha }}_{s}^{X}‐{\hat{\alpha }}_{l}^{X}}\right)$
CRIT. MSD ML	Critical MSD ML	${\hat{\alpha }}_{s}^{X}\times \text{CRIT}.\;\text{TIME}\;\text{ML}+{\hat{\beta }}_{s}^{X}$

### 3.5 Stochastic variables

The variables of this category are derived from stochastic models of the COP. The descriptors presented originate from the seminal work of Collins and De Luca (1993), which introduced the idea of the SDA.

#### 3.5.1 Stabilogram diffusion analysis

In Collins and De Luca (1993), the authors have suggested that the COP quadratic displacement is similar to the one of a fractional Brownian motion with two regimes. This claim was based on the analysis of the mean square displacement (MSD) defined as follows:

Definition 5 (MSD) For any 0 ≤ Δ*t *≤ Δ*t_N_* = T/3, the mean square displacement of the COP along the ML axis on the time interval Δ*t* is defined as.

$${\text{MSD}}^{X}\left(\Delta t\right)=\frac{1}{N‐F_{s}\Delta t}\sum_{n=1}^{N‐F_{s}\Delta t}{\left(X_{n+F_{s}\Delta t}‐X_{n}\right)}^{2}$$

This function can be similarly defined for the AP axis with *Y*.

**FIGURE 6:**
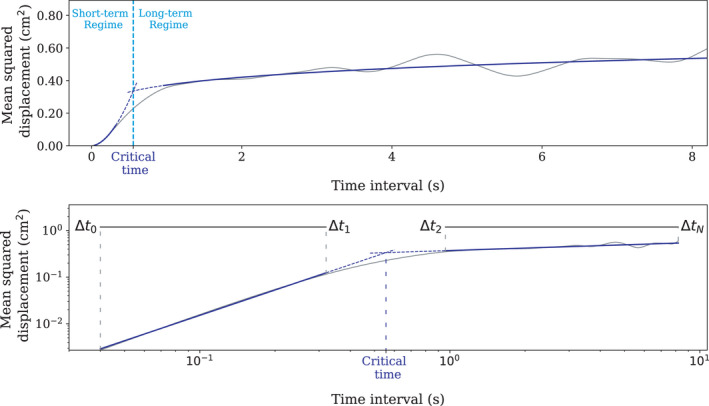
Example of stabilogram diffusion analysis and parameters estimation in each regime. The fitted functions in each region are drawn in blue. (Top) Curve of the MSD as a function of the time interval. (Bottom) Curve of the MSD as a function of the time interval on a logarithmic scale and intervals of time used for the estimation of the linear functions in each region

The constraint ∆*t* ≤ T/3 limits the definition of the MSD to time intervals shorter than one third of the total duration, a necessary restriction to avoid unreliable results (Collins & De Luca, 1993). In their work, Collins and De Luca (1993) have noted that there exists a critical time ∆*t_c_* such that the curve of the MSD variations with respect to ∆*t* (called diffusion plot) can be split into two regions with very different behaviors: a short‐term region (∆*t* ≤ ∆*t_c_*) and a long‐term region (∆*t* ≥ ∆*t_c_*) (see Figure 6). The short‐term and long‐term regions are the expression of different behaviors of the dynamic on different time scales: on short time scales, the system exhibits persistence, that is, positive correlation between successive displacements, and on longer time scales, the dynamic is anti‐persistent, meaning that the successive displacements are negatively correlated (Collins & De Luca, 1993). Different interpretations have been made following this observation. Collins and De Luca (1993) have claimed that it was the result of two different postural control regimes: on short time scales, the system evolves in open‐loop, whereas on longer time scales, control is activated and produces postural adjustments. This conclusion has been however refuted by several authors, with the argument that a closed‐loop continuous control model could reproduce similar patterns of the diffusion plot (Peterka, 2000). Several control models have been proposed to explain the phenomenon and there is no consensus on the true model of control which governs posture stabilization (Collins & De Luca, 1993; Chow & Collins, 1995; Delignières et al., 2011; Peterka, 2000). These short‐term and long‐term regions can be characterized through the estimation of the parameters in a two regimes model of the *MSD*. For this purpose, we use the single model formulation proposed by Chiari et al. (2000):

$${\text{MSD}}^{X}\left(\Delta t\right)=\left\{\begin{array}{c} D_{s}\Delta t^{2H_{s}}\;\text{for}\;\Delta t\leq \Delta t_{c}\left(\text{short}‐\text{term}\right)\\ D_{l}\Delta t^{2H_{l}}\;\text{for}\;\Delta t\geq \Delta t_{c}\left(\text{long}‐\text{term}\right) \end{array}\right.$$

where *H_s_* and *H_A_* are the short‐ and long‐term scaling exponents and *D_s_* and *D_A_* can be seen as short‐ and long‐term diffusion coefficients. Note that the model and the computation technique proposed in Chiari et al. (2000) are not exactly the same as the one formerly introduced in Collins and De Luca (1993), therefore the resulting features are not directly comparable with the previous ones. However, the general interpretation of the variables remains similar.

#### 3.5.2 Diffusion and scaling coefficients

For parameters estimation, the two regimes (short term and long term) of the MSD are approximated by two linear functions of the time interval on a logarithmic scale:

$$\ln\;{MSD}^{X}\left(\Delta t\right)=\left\{\begin{array}{c} \alpha_{s}^{X}\ln\left(\Delta t\right)+\beta_{s}^{X}\;\;\text{for}\;\;\Delta t\in \left[\Delta t_{0},\;\Delta t_{1}\right]\;\left(1\right)\;\mathrm{short}‐\mathrm{term}\\ \alpha_{l}^{X}\ln\left(\Delta t\right)+\beta_{l}^{X}\;\;\text{for}\;\;\Delta t\in \left[\Delta t_{2},\;\Delta t_{N}\right]\;\left(2\right)\;\mathrm{long}‐\mathrm{term} \end{array}\right.$$

If this model fitted perfectly the data, we could directly search for the time ∆*t_c_* which separates the diffusion plot into two different linear regions. However, as stated in Chiari et al. (2000), there exists for some trajectories a transition region in the MSD curve which is not well fitted by a linear model. For this reason the short‐term regime is estimated on a first region (∆*t*_0_, ∆*t*_1_) where ∆*t*_0_ = 1/F_s_ and ∆*t*_1_ is defined in the range (0.3 s, 2.5 s) as the highest time stamp which minimizes the root‐mean‐square‐error (RMSE) in the Ordinary Least Square (OLS) fit of the model **(1)**, then the long‐term regime is estimated on a second region (∆*t*_2_, ∆*t_N_*) where ∆*t_N_* = T/3 and ∆*t*_2_ is defined in the range (0.3 s, 2.5 s) as the highest time stamp which minimizes the mean square error in the OLS fit of the model **(2)**. An illustration of this estimation can be found in Figure 6.

Let ${\hat{\alpha }}_{s}^{X}$, ${\hat{\alpha }}_{l}^{X}$, ${\hat{\beta }}_{s}^{X},$ and ${\hat{\beta }}_{l}^{X}$ denote the OLS estimator of $\alpha_{s}^{X}$, $\alpha_{l}^{X}$, $\beta_{s}^{X},$ and $\beta_{l}^{X}$, respectively. Then:

$$\text{SHORT - TERM}\;\text{DIFF}.\;\text{ML}\;\exp\left({\hat{\alpha }}_{s}^{X}\right)$$

$$\text{LONG - TERM}\;\text{DIFF}.\;\text{ML}\;\exp\left({\hat{\alpha }}_{l}^{X}\right)$$

$$\text{SHORT - TERM}\;\text{SCAL}.\;\text{ML}\;\frac{{\hat{\beta }}_{s}^{X}}{2}$$

$$\text{LONG - TERM}\;\text{SCAL}.\;\text{ML}\;\frac{{\hat{\beta }}_{l}^{X}}{2}$$

These indices are similarly defined for the AP axis through replacing *X* by *Y*.

In other words, the SDA models the CoP behavior on short‐ and long‐term scales as two distinct stochastic processes. On the one hand, the diffusion coefficients are interpreted as the level of stochastic activity of the process in the two control regimes, along the mediolateral axis and the anteroposterior axis (Collins & De Luca, 1993; Melzer et al., 2010). The short‐term diffusion coefficient has been shown to differ significantly between individuals who sustained injuries after falls compared to non‐fallers and fallers without injuries (Kurz et al., 2013). On the other hand, the scaling coefficients are thought to quantify the correlation of the increments of the process in its persistent (short term) and its anti‐persistent (long term) regimes, along the mediolateral axis and the anteroposterior axis. In practice, the scaling coefficients generally appear to satisfy *H_s_* ≥ 1/2 and *H_l_* ≤ 1/2. Consequently, the short‐term increments are considered to be positively correlated and the long‐term increments are negatively correlated (Collins & De Luca, 1993). The long‐term scaling coefficient has been shown to significantly differ between young individuals and elderly (Muir et al., 2013), and could be impacted by muscular fatigue (Corbeil et al., 2003).

#### 3.5.3 Critical time and critical MSD

The critical time interval *δ_c_* is estimated as the value of *δ* for which the two linear functions in the logarithmic scale, **(1)** (short term) and **(2)** (long term), intersect. The critical mean square displacement is defined as the ordinate of the critical point, that is, the value of the linear approximation at the critical time interval (Melzer et al., 2010). It represents the mean quadratic displacement covered in the critical time interval, that is, in a period of persistence. While these variables differ significantly between fallers and non‐fallers in Tuunainen et al. (2014) and between individuals who sustained injuries after falls compared to non‐fallers and fallers without injuries in Kurz et al. (2013), previous works have shown that these variables 646 have low reliability (Qiu & Xiong, 2015). Moreover, these variables are uniquely derived from the other SDA 647 parameters and therefore with these additional features the model is not parsimonious (Chiari et al., 2000). ${\hat{\alpha }}_{s}^{X}$, ${\hat{\alpha }}_{l}^{X}$, ${\hat{\beta }}_{s}^{X},$ and ${\hat{\beta }}_{l}^{X}$ denote the OLS estimator of $\alpha_{s}^{X}$, $\alpha_{l}^{X}$, $\beta_{s}^{X},$ and $\beta_{l}^{X}$, respectively. Then:

$$\text{CRIT}.\;\text{TIME}\;\text{ML}\;\exp\left(\frac{{\hat{\beta }}_{s}^{X}‐{\hat{\beta }}_{l}^{X}}{{\hat{\alpha }}_{s}^{X}‐{\hat{\alpha }}_{l}^{X}}\right)$$

Note that if the estimated critical time is larger than duration limit ∆*t*_N_, it is set at ∆*t*_N_. The critical mean square displacement is defined as.

$$\text{CRIT}.\;\text{MSD}\;\text{ML}\;{\hat{\alpha }}_{s}^{X}\;\times \;\text{CRIT}.\;\text{TIME}\;\text{ML}\;+\;{\hat{\beta }}_{s}^{X}$$

Critical time and critical MSD are similarly defined for the AP axis with *Y*.

### 3.6 Descriptive analysis

Average values of the different variables and their standard deviations are reported in Table 7. Variables that are strongly correlated with the total duration of the recording (such as the Length Over Area) are reported separately in the second part of the table. Interestingly, and despite the significant differences between the two datasets, the range of values obtained were comparable for most variables. However, there are still some noticeable differences, particularly for the variables PEAK VELOCITY ML, TOTAL POWER ML, ENERGY CONTENT BELOW 0.5HZ ML, and SHORT TIME DIFFUSION ML which show standard deviations higher than the means on the SmartCheck database. This is in line with previous works that have hypothesized that longer recording duration might be necessary for the proper evaluation of the per spectrum (Vieira et al., 2009b). Moreover, the standard deviation of the variables is generally higher in the recordings from our protocol, which might result from both the shorter recording duration and the more varied demographics.

**TABLE 7:** Distribution of COP variables. For each variable, average values and standard deviations are reported in each dataset. WBB dataset refers to the data from our experiment, recorded with the Wii Balance Board and Public dataset refers to the open‐access dataset of human balance (Santos & Duarte, 2016a). Duration sensitive variables refer to variables that are strongly dependent on the duration of the recording

	Mean ± *SD* (WBB dataset)	Mean ± *SD* (Public dataset)
Mean distance ML	0.31 ± 0.25	0.24 ± 0.10
Mean distance AP	0.53 ± 0.28	0.39 ± 0.19
Mean distance radius	0.68 ± 0.38	0.51 ± 0.22
Maximal distance ML	1.21 ± 0.98	0.94 ± 0.40
Maximal distance AP	1.89 ± 1.00	1.47 ± 0.65
Maximal distance radius	2.05 ± 1.16	1.58 ± 0.68
Rms ML	0.40 ± 0.31	0.30 ± 0.12
Rms AP	0.66 ± 0.35	0.49 ± 0.24
Rms radius	0.79 ± 0.44	0.59 ± 0.26
Amplitude ML	2.08 ± 1.67	1.67 ± 0.69
Amplitude AP	3.37 ± 1.79	2.64 ± 1.15
Amplitude ML AND AP	3.59 ± 2.03	2.79 ± 1.20
Quotient both direction ML AND AP	0.62 ± 0.29	0.66 ± 0.17
Planar deviation ML AND AP	0.79 ± 0.44	0.59 ± 0.26
Coefficient sway direction ML AND AP	0.01 ± 0.30	0.03 ± 0.20
Confidence ellipse area ML AND AP	6.01 ± 9.35	3.02 ± 3.32
Mean velocity ML	0.83 ± 0.68	0.50 ± 0.22
Mean velocity AP	1.60 ± 1.36	0.87 ± 0.39
Mean velocity ML AND AP	1.97 ± 1.60	1.10 ± 0.47
Sway area per second ML AND AP	0.48 ± 0.79	0.18 ± 0.20
Phase plane parameter ML	1.20 ± 1.03	0.75 ± 0.31
Phase plane parameter AP	2.23 ± 1.74	1.25 ± 0.55
Peak velocity pos SPD ML	1.04 ± 0.98	0.65 ± 0.32
Peak velocity neg SPD ML	1.05 ± 1.06	0.65 ± 0.33
Peak velocity all SPD ML	1.05 ± 1.02	0.65 ± 0.33
Peak velocity pos SPD AP	2.17 ± 2.12	1.19 ± 0.60
Peak velocity neg SPD AP	2.14 ± 1.95	1.20 ± 0.64
Peak velocity all SPD AP	2.16 ± 2.03	1.19 ± 0.62
Mean peak sway density	1.05 ± 0.71	1.84 ± 0.92
Mean distance peak sway density	0.59 ± 0.39	0.34 ± 0.20
Mean frequency ML	0.52 ± 0.21	0.39 ± 0.13
Mean frequency AP	0.56 ± 0.29	0.42 ± 0.15
Mean frequency ML AND AP	0.48 ± 0.22	0.37 ± 0.12
Total power ML	3.03 ± 8.22	2.14 ± 2.08
Total power AP	6.33 ± 8.52	5.66 ± 10.22
Power frequency 50 ML	0.42 ± 0.13	0.43 ± 0.14
Power frequency 50 AP	0.37 ± 0.18	0.42 ± 0.13
Power frequency 95 ML	1.16 ± 0.42	1.09 ± 0.23
Power frequency 95 AP	1.33 ± 0.56	1.23 ± 0.24
Frequency mode ML	0.32 ± 0.17	0.33 ± 0.18
Frequency mode AP	0.25 ± 0.19	0.27 ± 0.14
Centroid frequency ML	0.65 ± 0.18	0.61 ± 0.14
Centroid frequency AP	0.69 ± 0.25	0.66 ± 0.14
Frequency dispersion ML	0.61 ± 0.07	0.56 ± 0.06
Frequency dispersion AP	0.65 ± 0.07	0.60 ± 0.05
Energy content below 05 ML	2.23 ± 7.21	1.36 ± 1.75
Energy content below 05 AP	4.23 ± 5.71	3.67 ± 8.47
Energy content 05 2 ML	0.75 ± 1.27	0.76 ± 0.90
Energy content 05 2 AP	1.83 ± 3.46	1.93 ± 2.23
Energy content above 2 ML	0.05 ± 0.24	0.01 ± 0.01
Energy content above 2 AP	0.26 ± 1.53	0.05 ± 0.07
Frequency quotient ML	0.02 ± 0.02	0.01 ± 0.00
Frequency quotient AP	0.03 ± 0.06	0.01 ± 0.01
Short time diffusion ML	0.72 ± 1.44	0.32 ± 0.34
Long time diffusion ML	0.36 ± 1.10	0.09 ± 0.14
Critical time ML	0.54 ± 0.74	0.41 ± 0.22
Critical displacement ML	0.31 ± 1.05	0.07 ± 0.14
Short time scaling ML	0.83 ± 0.07	0.90 ± 0.03
Long time scaling ML	0.17 ± 0.19	0.19 ± 0.10
Short time diffusion AP	1.72 ± 2.53	0.80 ± 1.03
Long time diffusion AP	0.88 ± 1.19	0.26 ± 0.59
Critical time AP	0.68 ± 0.47	0.43 ± 0.24
Critical displacement AP	0.81 ± 1.17	0.22 ± 0.58
Short time scaling AP	0.81 ± 0.10	0.88 ± 0.03
Long time scaling AP	0.08 ± 0.18	0.18 ± 0.12

Duration sensitive variables	Mean ± *SD* (WBB dataset)	Mean ± *SD* (Public dataset)
LFS ML AND AP	14.49 ± 9.52	31.26 ± 14.83
Fractal dimension ML AND AP	1.88 ± 0.23	1.98 ± 0.15
Zero‐crossing SPD ML	127.43 ± 34.16	195.47 ± 32.94
Zero‐crossing SPD AP	113.84 ± 31.43	200.08 ± 39.55

## 4 DISCUSSION

The main objective of this review is to present the variables calculated from the stabilogram that are most commonly used in the analysis of balance in elderly participants prone or not to fall. The rationale of this approach is to propose a common framework for the analysis of COP displacements by presenting together the calculation methods and the values obtained on two different databases. In order to provide an explicit corpus, we relied on a recent systematic review with published methodology and broad selection criteria for the variables. The results of 70 variables are presented for two groups of participants aged 60 and over, with and without a history of falls. The means and standard deviations thus obtained make it possible to appreciate the homogeneity of the values despite significant differences in the recording protocols. The first protocol corresponds to a methodology easily applicable in routine consultations, while the other is more in line with the metrological standards of posturography.

### 4.1 Aging and postural control

Falls in the older population are multifactorial in nature as they include socio‐economic and environmental elements in addition to biomedical factors. By providing a quantification of motor control in the elderly people, static posturography could help to determine a balance semiology (Nardone & Schieppati, 2010), especially for the most fragile people. This is particularly true since age‐related sensorimotor alterations can impact motor functions and increase the risk of falling (Ambrose et al., 2015). Static balance is controlled in a complex way by different sensory (visual, vestibular, proprioceptive, and tactile) and neuromotor systems (involving both sensory integration and movement planning to cortical control of standing and spinal reflex action resulting in changes in joint stiffness and damping; Goodman & Tremblay, 2021; Kang et al., 2013; Winter et al., 1998). Older people show altered motor strategies compared to young and healthy people, either for balance maintenance tasks or postural anticipation in the face of destabilization (Garcez et al., 2021; Woollacott & Manchester, 1993). But, in addition to the difficulty of studying the interactions between these systems and their actions in posture maintenance, there is a lack of interpretability of the COP variables (Palmieri et al., 2002), which is enhanced by the diversity of methods for calculating them. Finally, the choice of variables is difficult to justify from a physiological point of view (Chaudhry et al., 2011).

In the recent years, numerous methods have been proposed to analyze the trajectory of the COP, in order to investigate the differences betwe en elderly fallers and non‐fallers, as presented in our previous systematic review (Quijoux et al., 2020). At the same time, the univariate analysis of postural variables provides limited information on the physiological causes of falls (Duarte & Freitas, 2010). This has encouraged the multiplication of variables, as it may be necessary to analyze all the components of the stabilogram—in a particular axis and in two dimensions—to fully capture the COP dynamics and the age‐related motor adaptations (Bargiotas et al., 2018). Indeed, age‐related decline in postural control is not uniform, which is understandable given the various anatomical structures that may be affected (Shaffer & Harrison, 2007).

Distal myelin fibers and sensory receptors are affected by senescence and sedentary life, leading to impaired proprioception, particularly in the hips, knees, and ankles (Horak et al., 1989; Robbins et al., 1995), as well as loss of touch discrimination (Perry, 2006), with a potential predominance in the distal joints of the lower limb (Pickard et al., 2003; Shaffer & Harrison, 2007). At the neuromuscular level, all the contractile properties of the muscles are impacted (Liu et al., 2005), notably by the reduction in the vascular feeding system and thus, in the number of muscle fibers, their volume and their contractibility. Presynaptic inhibition of Ia afferents, which plays a role in leg muscle contractility, is more favored in the elderly when sensory and somesthetic afferents are reduced (Baudry & Duchateau, 2012). This type of neuromuscular alteration could partly explain the adoption of a leg muscle co‐contraction strategy in the elderly (Papegaaij & Hortobágyi, 2017). This co‐contraction may reduce the exploitation of proprioceptive afferents from the mechanoreceptors (Baudry, 2016; Benjuya et al., 2004; Craig et al., 2016) and the efficiency of the muscular efferents in the segmental control of balance (Finley et al., 2012; Nelson‐Wong et al., 2012). A significant correlation between the increase in co‐contraction measured in the elderly and the increase in MAX AP was found, whereas it was absent in young adults (Baudry & Duchateau, 2012). As a result, studies agree that an overall shift in balance control from spinal to supraspinal levels occurs in older adults, in line with what is found in healthy subjects when proprioceptive afferents decrease (Alizadehsaravi et al., 2020). Given the diversity of disorders affecting the elderly, a bilateral alteration of the vestibular system could lead to an increase in the values of the COP variables, as seen on SWAY LENGTH (Mbongo et al., 2005). When visual inputs are altered (with the use of a moving target), there is an increase in the contribution of the knee and hip joints, which correlate with an increase in COP variables in the elderly people (Freitas & Duarte, 2012). An increase in the amplitude of displacement suggests a decrease in the ability to maintain a stable upright position, but the diversity of results obtained for positional and dynamic variables led Palmieri et al. to minimize their clinical interpretation (Palmieri et al., 2002). Dynamic, frequency, and stochastic variables could provide complementary and clinically relevant information. Although more studies are needed before concluding on their physiological interpretation, we note that biomechanical modeling has shown a negative correlation between the supposed stiffness of the system and mean frequency and MEAN VELOCITY, but positive with CRITIAL TIME (Maurer & Peterka, 2005).

### 4.2 Feature classification

To the best of our knowledge, the classification of posturographic variables that is introduced in this study is new and differs from previous classification paradigms. Duarte and Freitas (2010) used a classification which distinguishes the descriptors resulting from a *structural analysis*—that is, which aim to explain the control postural commands through the behavior of the COP, with sway density models or stochastic models—from other variables. In Prieto et al. (1996), four categories of descriptors were proposed: (1) time‐domain distance measures, (2) time‐domain area measures, (3) hybrid measures, and (4) frequency‐domain measures. The first class includes features associated with either the displacement of the COP from the average, or the velocity; the second gathers geometric approximations of the surface of the COP; the third includes combinations of distance measures (Prieto et al., 1996), which have been considered by others as dimensionless features (Qiu & Xiong, 2015); the fourth contains variables related to the analysis of the power spectral density of the COP trajectory, usually obtained through Fast Fourier Transformation (FFT). Our classification, while similar to the one proposed in Prieto et al. (1996), presents two major differences. First, since the work of Prieto et al. (1996), popular stochastic models have been developed (Collins & De Luca, 1993; Duarte & Freitas, 2010; Qiu & Xiong, 2015). Hence we introduce a fourth category of variables, called stochastic descriptors, which includes the features derived from stochastic‐based models of the COP. Second, we choose to regroup the non‐stochastic, non‐frequency derived descriptors into positional and dynamic classes. Importantly, this classification originated from signal processing concepts, and its main purpose was to ease the reading of this study.

### 4.3 Variables reliability

The reliability of stabilogram variables depends on several factors. The variation in the values of the posturographic variables recorded on the force platform reflect the participation of the muscles involved in maintaining balance and the contribution of the joints to postural oscillations. Feet placement could also modify postural strategy in older population (Chiari et al., 2002; Winter et al., 1996). For instance, when feet are joined, the ML displacements of the COP are mostly influenced by the hip adductors and abductors, whereas in the tandem position, movements in the ML direction are mostly related to the contractions of the invert and spurs muscles of the leg (Prince et al., 1995; Winter et al., 2003, 1996). In the upright, straight position, feet open up by 45^◦^ apart, the movement in the ML direction is a mix of hip and ankle strategies, whereas the AP displacements are under the dominance of the ankle muscles.

Anthropometric factors influencing posturographic variables include height, weight, maximum foot width, base of support area, and foot opening angle as the relevant biomechanical variables (Chiari et al., 2002). The authors note a significant dependence of gender for the SWAY LENGTH, in the AP direction with eyes open. This could be explained by higher “height” and “weight” in males, with which the variables are strongly positively correlated. As also mentioned by the authors, several ML variables, especially positional (MEAN DISTANCE ML, SWAY LENGTH ML, RMS ML, RANGE ML), dynamic variables (MEAN VELOCITY ML), and frequentist variables (TOTAL POWER, FREQ. DISP. ML), decrease while base of support increase, in eyes‐open condition. Few frequentist variables are positively correlated with the size of the base of support (50% POWER FREQ ML, 95% POWER FREQ, CENTROIDAL FREQ ML). At the same time, the foot opening angle could have only a marginal or no impact on the variable values during open‐eyes recordings. The maximum foot width showed a positive correlation for several frequentist variables but negative for the stochastic variables (notably SHORT TIME DIFFUSION COEFFICIENT, LONG TIME DIFFUSION COEFFICIENT, and SHORT TIME SCALING DIFFUSION). These results illustrate the impact of morphological factors and foot position on the variables that vary within each family. It should be noted that these results are based on a signal filtered at 8 Hz and downsampled at 20 Hz. Between sessions, posturographic variables have shown good reliability in the elderly people with the same experimental conditions (Li et al., 2016). Riemann et al. have shown a better reliability of the variables when the position of the feet was left at the participant's choice, also considered as comfortable (Riemann & Piersol, 2017). Imposing a standardized foot placement could lead to a change of the biomechanics of the lower limp by reducing the number of degrees of freedom and hence, modify the strategy adopted to maintain balance (Gibbons et al., 2019). Finally, the authors do not agree on a recommendation concerning the position of the feet and the width of the base of support, either by standardizing them or by leaving it to the subject's choice of comfort, to increase the reliability of the measurements (Riemann & Piersol, 2017; Ruhe et al., 2010).

The differences between the values reported in the literature may also be explained by differences in equipment, sampling frequency, preprocessing, and acquisition protocol (Carpenter, Frank, Winter, et al., 2001; Ruhe et al., 2010; Vieira et al., 2009b; Schmid et al., 2002). First, the sampling frequency varies greatly between studies. The sampling frequency seems to have a greater impact on frequency variables than on positional and dynamic variables. Rhea et al. add that a decrease in the sampling frequency (from 100 to 25 Hz) has a non‐significant impact on the nonlinear analyses to obtain the stochastic variables (Rhea et al., 2015). The reliability of the WBB, used in this study, has been widely studied in the literature and the authors generally conclude that it can be used to record balance (Abujaber et al., 2015; Bartlett et al., 2014; Clark et al., 2010; Severini et al., 2017). However, we would emphasize the need to correct the sampling frequency of this force platform and refer the readers to our previous work for more details (Audiffren & Contal, 2016).

Second, the differences between preprocessing strategies that can be found in the literature may alter the computation of the parameters (Schmid et al., 2002), in particular for the dynamic group, as they involve the derivative of the trajectory and are sensitive to the cut‐off frequency of applied filters. This led to the recommendation of a sampling frequency of 100 Hz and a cut‐off frequency of 10 Hz, in the absence of further studies (Ruhe et al., 2010).

Third, reliability may be affected by the acquisition protocol. It has been claimed that a sufficient recording duration, generally around 60 s, is required to obtain a robust estimation of the power spectral frequency (Vieira et al., 2009b). The dynamic variables could show a greater reliability as the recording time is increased, up to 90 s, and then the benefit would be less noticeable (Ruhe et al., 2010). However, the relevance of continuing the recordings beyond 60 s must be measured according to the population to be recorded because, on the one hand, good reliability has been obtained with dynamic and stochastic variables as early as 30 s (Caballero et al., 2015; Nagymáté et al., 2018) and the reproducibility of the variable measurements does not show the same dependence on the duration of recording according to the families of variables (Nejc et al., 2010), while on the other hand, proposing long recordings with several repetitions does not seem very feasible for measuring the balance in the clinical context, especially for extremely fragile people (Alsubaie et al., 2019). Additionally, many of the parameters, such as the MEAN VALUE, RMS, and all variables derived from the power spectral density analysis, are based on the assumption that the COP signal is stationary, which is generally not true (Strang et al., 2013). This could significantly impact the variability of the parameters (Carroll & Freedman, 1993).

This influence of individual factors, experimental conditions, and preprocessing methods on the values of the COP variables makes particularly essential studies reproducibility which could be eased by the use of standardized definitions and implementation of the posturographic variables.

### 4.4 Scope and limitations

This review focuses on the variables used to discriminate between elderly fallers and other older adults. However, in order to generalize the description of the variables, and more generally the mathematical requirements for calculating them, it was necessary to extend the search to the references of the articles, which made it possible to highlight the reliability of several indices as well as their variability according to age. This review does not take into account indices that can be used to distinguish between younger and older participants and as such cannot be described as comprehensive. Many other posturographic variables have been proposed to 819 assess the risk of falls in older people, either through measures of dynamic balance (Ringhof & Stein, 2018), or in correlation with clinical assessments of motor skills (Cheng et al., 2012; Karlsson & Frykberg, 2000), or because they are less commonly found in the literature, which did not fit the selection criteria of this review. Regarding the latter, we have not included variables based on biomechanical or other equilibrium modeling (Koltermann et al., 2020; McKee & Neale, 2019; Nicolai et al., 2021), as well as several other modelizations such as wavelet analyses (Chagdes et al., 2009), sample entropy analysis, and other associated entropies computations (Degani et al., 2017; Gow et al., 2015) or analyses based on Markov chains (Hur et al., 2012). To overcome these limitations, further literature reviews should be conducted in the future to explore the most recent methods that have been applied to the postural signals. This would require going beyond the variables used to discriminate between fallers and non‐fallers.

We only present the calculation methods here, but the search for correlations between the risk of falling and these posturographic variables and their exploitation for prevention purposes leads to selection processes. Several models could be considered to identify the most relevant variables in the assessment of fall risk, whether using a Poisson regression (Palumbo et al., 2015) or zero‐inflated models (Ullah et al., 2010) to describe the number of falls in a given time as well as other nonlinear approaches with a selection process of the multiple variables as it was recently performed in patients with Parkinson's disease (Bargiotas et al., 2021) or between healthy fallers and non‐fallers (Audiffren et al., 2016).

The presentation of the values on the basis of two different recording protocols, and the similarity of the results obtained for these two populations, should enable more homogeneity in future studies, while the link between the physiology of static balance and these posturographic variables remains to be clarified.

## 5 CONCLUSION

A review of the literature on the analysis of the characteristics of the COP for the discrimination of elderly people at high risk of falling revealed the lack of information concerning the methods of calculation of the posturographic variables used, as well as the lack of homogeneity and standardization between studies. By presenting a comprehensive glossary of calculation methods and a library of functions that is as clear and exhaustive as possible, this should facilitate reproducibility between studies. Comparison with future studies should also be made easier by providing a basis for comparing these variables for two different protocols of COP recording, in elderly participants, with or without a history of falls. The choice of the selection of variables among the growing number of possible methods of analysis of the COP trajectory should be explained, in particular to make explicit whether it is based on a statistical approach to reduce the dimensionality of the exploration or on habits that are the result of clinical experience and interpretability of the chosen variables. Furthermore, the exact definitions of the variables used should be detailed and it should be precised if these variables depend strongly on the standardization of foot placement or on the length of the recording. In addition, despite the similarities that we observed between the values obtained with two different protocols of quiet stance balance recorded on two separated samples of elderly people, it is advisable to follow the recommendations concerning recording duration (of at least 60 s with several repetitions), the sampling frequency (100 Hz and a cut‐off frequency of 10 Hz) and a standardization of the placement of the feet on the force platform (especially if the variables that depend on the base of support are used), when it is possible. Regarding the instructions, the positioning of the arms, generally alongside the body, the use of instructions to the participant such as to remain stable without moving or the addition of a visual target to facilitate standing at a distance of a few meters from the person should be indicated. These recommendations must take into account the feasibility of recording balance in a real environment, which does not necessarily permit this level of standardization depending on the equipment used, the space available, or the physical capacities of the elderly people being recorded, especially when their frailty leads to a high risk of falling, since these people are probably the ones who could benefit most from fine balance measurements. Future studies with a larger sample size and longitudinal follow‐up could further investigate the choice of a combination of postural variables, as well as the benefits of multidimensional analysis in elderly people.

## Footnotes

^1^
This definition is contested, see for example, (Gagey, 1999).
